# All‐Solution‐Processable Hybrid Photothermoelectric Sensors with Carbon Nanotube Absorbers and Bismuth Composite Electrodes for Nondestructive Testing

**DOI:** 10.1002/smsc.202400448

**Published:** 2025-02-20

**Authors:** Yuto Matsuzaki, Reiji Tadenuma, Yuto Aoshima, Minami Yamamoto, Leo Takai, Yukito Kon, Daiki Sakai, Norika Takahashi, Ryo Koshimizu, Qi Zhang, Naoko Hagiwara, Meiling Sun, Daiki Shikichi, Raito Ota, Sayaka Hirokawa, Yukio Kawano, Kou Li

**Affiliations:** ^1^ Faculty of Science and Engineering Chuo University 1‐13‐27 Kasuga, Bunkyo‐ku Tokyo 112‐8551 Japan; ^2^ Laboratory for Future Interdisciplinary Research of Science and Technology Tokyo Institute of Technology 2‐12‐1 Ookayama, Meguro‐ku Tokyo 152‐8552 Japan; ^3^ Department of Electrical and Electronic Engineering, School of Engineering Tokyo Institute of Technology 2‐12‐1 Ookayama, Meguro‐ku Tokyo 152‐8552 Japan; ^4^ National Institute of Informatics 2‐1‐2 Hitotsubashi, Chiyoda‐ku Tokyo 101‐8430 Japan; ^5^ Kanagawa Institute of Industrial Science and Technology 705‐1 Imaizumi, Ebina‐shi Kanagawa 243‐0435 Japan

**Keywords:** 3D imaging, broadband photomonitoring, nondestructive inspections, paste materials, photothermoelectric effects, thin‐film electronics

## Abstract

While photothermoelectric (PTE) sensor sheets are potentially suitable for testing applications, such as nondestructive material identifications in ultrabroad millimeter‐wave infrared bands, their device designs have primarily employed a single‐material channel. Herein, PTE sensor sheets generally combine photoinduced heating with associated thermoelectric (TE) conversion phenomena, and the employment of a single‐material channel regulates device operations by missing opportunities for fully utilizing their fundamental parameters. For this situation, this work develops all‐solution‐processable and freely coatable (paintable) hybrid PTE sensors by an effective combination of the channel structure with bismuth composite (Bi_com_) TE electrodes (Seebeck coefficient > 100 μV K^−1^) and efficient carbon nanotube film photothermal absorbers. This hybrid PTE sensor device stably forms its TE electrodes as easy‐to‐handle pastes of Bi_com_ material powders with high Seebeck coefficients by effectively employing conductive solvents and surfactants. Following these material and process preparations, the hybrid PTE sensor functions in ultrabroadband regions beyond the conventional detectors with comparable sensitivities to the existing narrowband devices in individual ranges and provides diverse optical measurement opportunities. Indeed, the easy‐to‐handle device fabrication process and advantageous photodetection performances of the hybrid PTE sensor demonstrate high usability for nondestructive testing applications (noncontact inspections, panoramic 3D camera monitoring, and portable device setups).

## Graphical abstract

Herein, potential of the photothermoelectric (PTE) sensor device is fully maximized by effectively combining carbon nanotube film‐based ultrabroadband absorbers and bismuth composite‐type sensitive thermoelectric‐converting electrodes. Such hybrid PTE sensors are advantageously available via mechanical coating processes or manual painting steps at high yield regardless of substrates and spatial deformations, by collectively handling respective constituent materials in ink or paste forms.

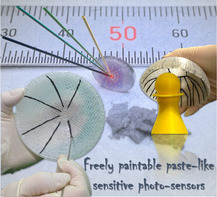

## 1 Introduction

In recent automation trends for social manufacturing and distribution sectors, nondestructive testing techniques play an indispensable role.^[^ ^1^, ^2^ ^]^ The major expected inspection targets are internal material identifications for 3D objects. This situation is mainly because most commodities and industrial products are composed of combining various 3D spatial positions and shapes of composite materials.^[^ ^3^, ^4^ ^]^ For the above situation, the use of longer‐wavelength photosensor sheets is effective as inspection devices.

In particular, the longer‐wavelength photosensor sheets potentially identify internal materials of various 3D objects regardless of shape and size configurations in a nondestructive manner. Here, the inherent optical properties of longer‐wavelength photoirradiation, such as millimeter‐wave (MMW),^[^ ^5^ ^]^ terahertz (THz),^[^ ^6^ ^]^ and infrared (IR),^[^ ^7^ ^]^ provide the above potentials. In general, MMW–IR irradiation exhibits noninvasive permeability to various nonmetallic materials.^[^ ^8^, ^9^ ^]^ Furthermore, transmittance values of nonmetallic materials to MMW–IR irradiation are specifically variable depending on compositions and wavelengths.^[^ ^10^, ^11^ ^]^ In other words, MMW–IR irradiation nondestructively identifies core constituent materials (e.g., glass, semiconductor, plastic, ceramic, and so on) of commodities and industrial products.

Following these optical advantages, the device design as thin‐film sensor sheets potentially facilitates freely attachable and flexibly designable inspection system configurations.^[^ ^12^, ^13^ ^]^ In particular, the above features provide omnidirectional views without blind spots by comprehensively surrounding 3D inspected objects with sensor sheets.^[^ ^14^ ^]^ Such an approach advantageously avoids degradations of optical information at side, rear, curvature, and bumpy structures of inspection targets.^[^ ^15^ ^]^

For developing such longer‐wavelength optical sensor sheets, material and device design strategies based on the photothermoelectric (PTE) effect play a leading role.^[^ ^16^, ^17^ ^]^ Here, the PTE effect synergizes two different energy harvesting phenomena (photoabsorption‐induced heating and the associated thermoelectric (TE) conversion).^[^ ^18^ ^]^ Following these physical mechanisms, the PTE effect detects ultrabroadband photoirradiation at room‐temperature conditions in zero‐bias‐voltage operations.^[^ ^19^ ^]^ In other words, PTE sensors adequately handle longer‐wavelength photoirradiation with compact thin‐film sheet structures themselves (free from bulky cooling and power source units).^[^ ^20^ ^]^

Despite these advantageous optical properties, conventional design strategies on PTE sensor sheets are still insufficient for device operations with high usability. In particular, the conventional PTE sensor designs mainly have focused on suppressing noise signals for sensitive photodetection operations.^[^ ^21^, ^22^ ^]^ In other words, the typical conventional PTE sensor sheet provides responses in approximately hundreds of μV—a few mV ranges against external photoirradiation.^[^ ^23^, ^24^ ^]^ For example, this situation potentially hinders coupling compact readout circuits or modules with PTE sensor sheets for on‐site nondestructive testing device applications. This is mainly because compact wireless circuits essential for on‐site mobile experimental setups typically exhibit readout resolution ranges around a single‐digit mV.^[^ ^25^, ^26^ ^]^

In these examples, the utilization of representative thin‐film materials with Seebeck coefficients in tens of μV K^−1^ ranges for typical PTE sensor sheet devices leads to the above fatal mismatch. Here, typical thin‐film PTE sheet materials include carbon nanotube (CNT) films,^[^ ^27^ ^]^ graphene,^[^ ^28^ ^]^ conducting polymers (e.g., poly(3,4‐ethylenedioxythiophene)‐poly(styrenesulfonate); PEDOT:PSS),^[^ ^29^ ^]^ black phosphorus,^[^ ^30^ ^]^ and transition metal dichalcogenides (e.g., MoS_2_ and SnSe).^[^ ^31^ ^]^ These thin‐film materials provide freely attachable configurations and omnidirectional views without blind spots as sheet devices,^[^ ^32^ ^]^ and their inherent diverse optical properties (e.g., broadband,^[^ ^33^ ^]^ selectivity,^[^ ^34^ ^]^ and transparency^[^ ^35^ ^]^) enrich photosensing functionalities. The typical conventional PTE sensor sheets mostly comprise these single kinds of thin‐film materials. Indeed, such single‐material configurations attenuate noise signals in photodetection responses by controlling the Fermi level,^[^ ^36^ ^]^ channel morphologies,^[^ ^37^ ^]^ and so on. Whereas, the PTE effect (the operating mechanism of the sensor sheet) inherently requires both optical and TE characteristics of device materials regarding photodetection response signal intensities.^[^ ^16^ ^]^ In other words, the PTE sensor sheet design for various device applications principally needs to satisfy synergistic combinations of optical materials (for defining operation bands) and TE layers (for governing response intensities). In particular, implementations of TE layers with higher Seebeck coefficients into PTE sensor sheets potentially facilitate device operations at even over two digits mV photodetection response signals, as an example. However, efforts are still insufficient regarding the development of hybrid PTE sensor sheets by synergizing multiple materials with optimized respective optical and TE properties.

To this end, this work effectively separates the PTE sensor structure into TE conversion electrodes of bismuth composite (Bi_com_) materials (Seebeck coefficients over 100 μV K^−1^) and a CNT film photothermal absorber (Figure S1, Supporting Information). Here, Bi_com_ materials such as bismuth telluride^[^ ^38^ ^]^ and bismuth antimony^[^ ^39^ ^]^ play a leading role in energy harvesting devices and heat‐waste recycling systems with their inherently advantageous TE conversion properties.^[^ ^40^, ^41^ ^]^ Whereas, the utilization of those Bi_com_ materials mostly employs bulky configurations such as chips^[^ ^42^ ^]^ and pellets^[^ ^43^ ^]^ and is insufficient for flexibly designable integrations into thin‐film PTE sensor sheet devices for nondestructive testing applications. The above situation hinders opportunities for the employment of promising Bi_com_ materials in ultrabroadband (MMW–IR) PTE sensor sheet devices. In contrast, this hybrid PTE sensor stably forms Bi_com_ materials as easy‐to‐handle electrode pastes by effectively employing conductive solvents and surfactants with inherently ink‐formed CNT film absorbers. This work developed all‐solution‐processable and freely coatable (paintable) hybrid PTE sensors by combining the paste treatment of Bi_com_ TE electrode materials with ink‐based CNT film photothermal absorbent channels. Following these material and process preparations, the hybrid PTE sensors proposed in this work function in ultrabroadband regions beyond the conventional detectors with comparable photosensitivities to previous narrowband devices in individual ranges and offer diverse optical measurement opportunities. Indeed, the easy‐to‐handle device fabrication process and advantageous photodetection performances of the hybrid PTE sensor demonstrate high usability for nondestructive testing applications (multiple‐pixel integrations, 3D panoramic painting, and compact portable setups).

## 2 Result and Discussion

### 2.1 Material Properties of the Hybrid PTE Sensor

**Figure 1:**
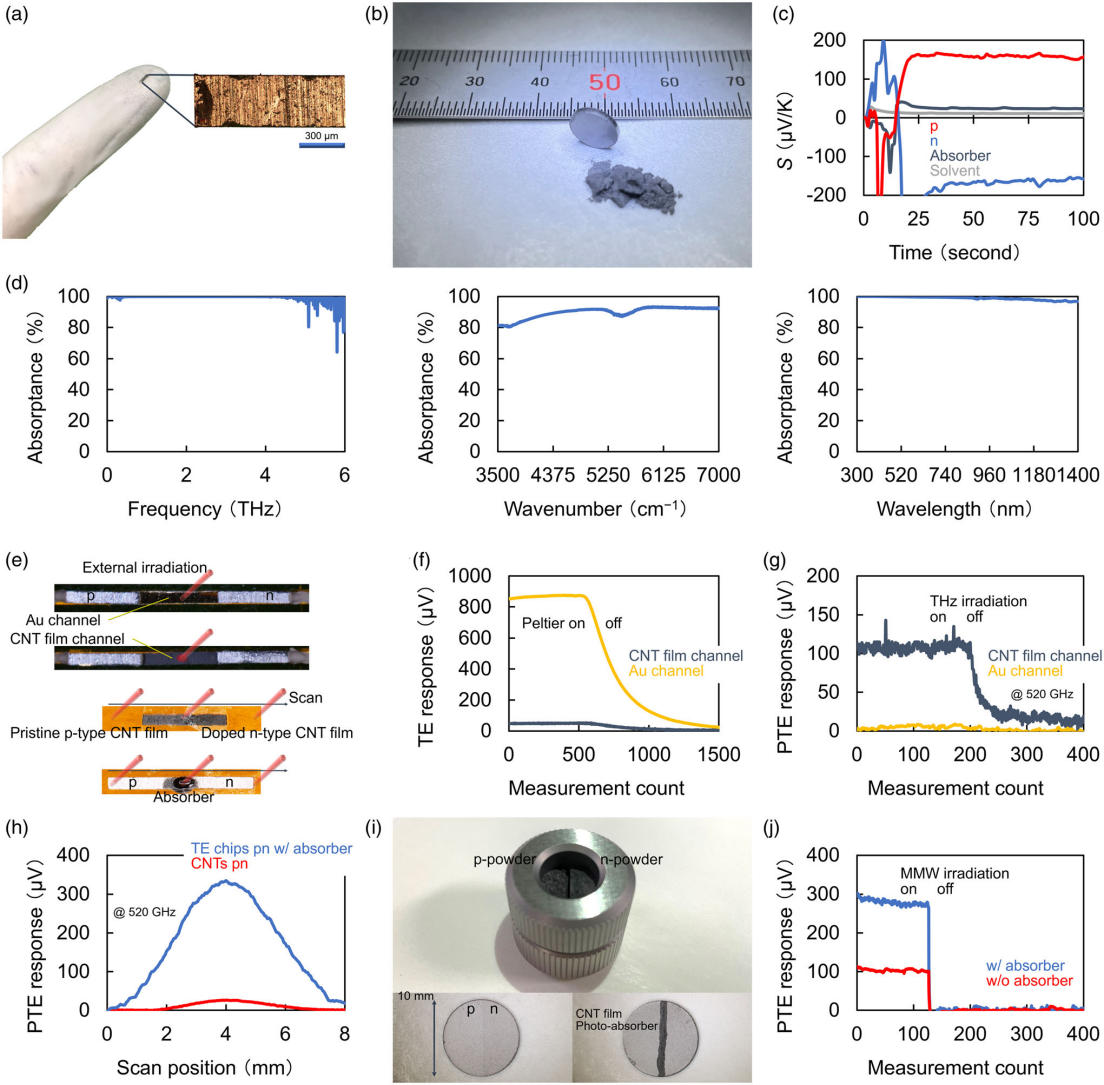
Fundamental PTE properties of device materials in this work. a,b) Photographs of Bi_com_ materials. c) Seebeck coefficient of each material. d) Optical properties of the CNT film photoabsorption channel in ultrabroad MMW–vis bands. e) Principle comparison of TE and PTE conversions. f,g) Experimental comparison of the TE (f) and PTE (g) conversions in (e). h) Comparison of PTE conversions in the bottom half of (e). i) Photograph of p–n junction Bi_com_ pellets. j) Change in PTE responses of samples (i) with and without the CNT film photoabsorption channel.

The purpose of this work is the development of hybrid sensor sheet structures comprising CNT film absorbers (for photoinduced heating) and Bi_com_ electrodes (for TE conversion) to fully maximize the inherent potentials of the PTE effect, toward realizing ubiquitous nondestructive inspection tools as easy‐to‐operate paste‐like forms. As the first step in designing the hybrid PTE sensor structure, this work evaluates the fundamental properties and features of the respective constituent materials of the device. **Figure** **1**a,b shows typical handling forms (chips, powder, and bulk) of Bi_com_ materials. The presenting hybrid sensor sheet in this work employs these Bi_com_ materials as p–n junction TE conversion electrodes. This device also performs highly efficient PTE conversions through the structure with CNT film channels (for photoabsorption and heating) on the p–n junction. Figure 1c shows the Seebeck coefficients of the above device materials. The obtained result indicates that the absolute values of the Seebeck coefficients of p‐ and n‐type Bi_com_ materials are over seven times higher than that of the CNT film. From the above situation, this device shows dominant TE conversions at the Bi_com_ electrodes. “Device materials” and “Seebeck coefficient measurement” in the Section 4 describe the detailed conditions of the device materials. Whereas, the CNT film channels exhibit highly efficient (over 80% throughout) photoabsorption properties in ultrabroad MMW–vis bands (Figure 1d, also see “Spectroscopy” in the Section 4). Here, note that the spectral absorption of the CNT film channel exceeds the measurement ranges of the equipment. Indeed, this work contains optical measurements in the following sections with wavelength (e.g., *λ*: 4.33 μm) out of scanning ranges in Figure 1d. This advantage develops typical conventional TE conversion structures (with p–n junction Bi_com_ materials) into functional optical techniques assisted by photoinduced heating.

Figure 1e shows an example of the earlier concept. For a deeper understanding of the following device behaviors, “TE and PTE effect” in the Section 4 briefly describes sensor operation characteristics. The first example is a replacement of the conventional metal heat‐conducting material to the CNT film as the channel layer of TE conversion structures. This work prepared Au and CNT thin films as the channel materials for p–n junction Bi_com_ electrodes. The Au channel presents thermal conductivity over 19 times higher than that of CNT films,^[^ ^36^ ^]^ and the employment of the former enables dominant TE conversions (Figure 1f). For TE conversion evaluations, this work installed a micro‐Peltier module on each channel (Figure 1e). Then, the module applied 3 °C heating to the channel. Whereas, the Au channel functions as a reflective layer against photoirradiation. This situation suppresses supplying photoabsorption‐induced heating to Bi_com_ electrodes. Indeed, the employment of the Au channel is insufficient for the PTE sensor structure, as shown in Figure 1g. Contrary to TE conversions, the CNT film channel facilitates photodetection operations with p–n junction Bi_com_ electrodes via the PTE effect. This work sets THz irradiation to each channel (Au/CNTs thin‐film structures), exhibiting PTE responses over 14 times higher in the latter. These hybrid conversions facilitate advantageous photodetection operations over typical conventional p–n junction CNT film sensor structures.^[^ ^44^, ^45^ ^]^ This work prepared different p–n junction PTE sensors: CNT film‐ and Bi_com_ electrodes‐based structures (bottom half of Figure 1e). As shown in the previous works, p–n junctions generally exhibit the highest response sensitivities across PTE sensor structures and function as photodetection interfaces.^[^ ^46^ ^]^ Figure 1h scans THz irradiation along the length (thermal diffusion) direction of prepared PTE sensors, and a timing of 4 mm on the horizontal axis of the graph corresponds to the spatial position of the p–n junction. The obtained results show a 13‐fold higher photodetection response of the hybrid PTE sensor to the p–n junction structure of the CNT film itself. This situation effectively demonstrates the significance of advantageous Seebeck coefficients (Figure 1c) of the Bi_com_ over CNT films. Here, “Signal readout” and “Photosources” in the Section 4 briefly describe specific setups for these optical evaluations.

Simultaneously, Figure 1i,j emphasizes the necessity for CNT film photothermal channels in p–n junction Bi_com_ structures. This work fabricated direct‐contacted junction structures with respective p‐ and n‐type Bi_com_ layers by a powder pressure forming method. For two prepared pellets, this experiment formed the CNT film photothermal channel on the p–n junction of one sample and kept the remaining type as the initial condition. For these samples, the obtained results compare PTE responses against MMW irradiation. The former sample shows PTE responses approximately three times higher than that of the latter, assisted by the efficient photoinduced heating of the CNT film channel. Figure S2, Supporting Information, describes detailed fabrication conditions of p–n junction Bi_com_ pellets. By incorporating these findings (from metal to CNT: TE conversion module channel, from single material to composites: PTE sensor structure), the hybrid device design proposed in this work facilitates p–n junction Bi_com_ electrodes‐based sensitive photodetection operations.

### 2.2 Paste‐Like Design for Bi_com_ TE Converting Electrodes of the Hybrid PTE Sensor

**Figure 2:**
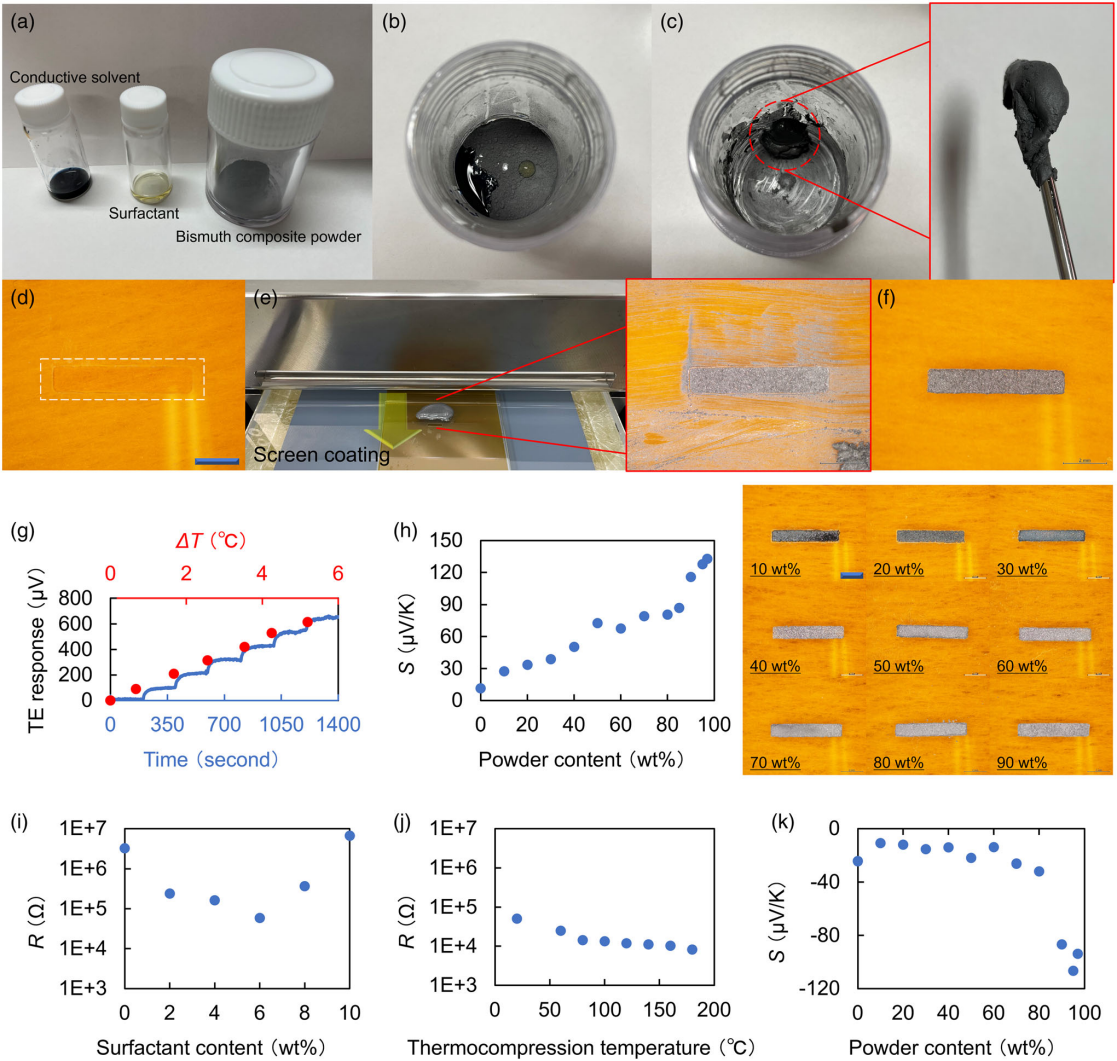
Fabrication, coating, and fundamental evaluation of Bi_com_ electrode pastes. a–c) Bi_com_ paste fabrication process. d–f) Screen‐coating process of the pastes. Scale bar: 2 mm. g) TE conversion with the p‐type Bi_com_ paste (90% powder content). h) Change in the Seebeck coefficient of the p‐type paste depending on Bi_com_ powder content. Scale bar: 2 mm. i,j) Changes in the electrical resistance values against surfactant content of the paste (i) and thermocompression temperature after coating (j). Bi_com_ powder content: 90% (i,j). k) Change in the Seebeck coefficient of the n‐type paste depending on Bi_com_ powder content.

To make the most of the above performances of Bi_com_ materials as TE electrodes in thin‐film PTE sensor sheet structures, their paste treatments from the inherent bulky configurations are indispensable. **Figure** **2**a–c shows Bi_com_ paste fabrication processes. In this paste fabrication, the constituent materials are as follows: conductive solvent, surfactant liquid, and Bi_com_ powder (see “Device materials” in the Section 4). This work mixes the above materials via magnetic stirring. Here, the conductive solvent plays a liquefying role in the Bi_com_ powder.^[^ ^47^ ^]^ Furthermore, the surfactant relaxes agglomerations of the Bi_com_ powder in the paste, leading to improved coating and printing of them.^[^ ^48^ ^]^ This work employs these pastes as screen‐coating inks.^[^ ^49^ ^]^ Figure 2d–f shows the screen‐coating process of the pastes. For the screen coating of the Bi_com_ pastes, this work first prepared laser‐processed thin‐film masks, a coating substrate, and a supporting glass (Figure 2d). The coating substrate was a double‐sided adhesive polyimide (PI) film. One side of the film was firmly attached to the coating mask and the other side to the coating substrate (see “Device fabrication” in Section 4). The experimental procedure: setting the substrate on the supporting glass, firmly attaching the mask to the substrate, dropping ink materials on the mask, applying the ink across the entire mask area via mechanical pushing of the screen bar, and drying (Figure 2e). The depositions of Bi_com_ paste are completed by mask detachment. The proposed method selectively forms TE conversion electrodes in areas inside the window and removes the excessively applied pastes during screen coating with the mask detachment (Figure 2f). For the above consecutive process of Bi_com_ paste preparations and formations, Figure S3, Supporting Information, introduces detailed experimental conditions in a flowchart manner. In these paste coatings, Figure S4, Supporting Information, evaluates line and space values as the fundamental printing accuracy.

To understand the fundamental features of Bi_com_ pastes, Figure 2g demonstrates applying a temperature difference to the deposited sample. The obtained results show voltage response signals linearly proportional to the temperature difference in the sample. This situation indicates that the Bi_com_ electrodes deposited from the pastes properly perform TE conversions. Relatedly, Bi_com_ powder contents during paste fabrications dominantly control the Seebeck coefficient of the above TE electrode. Figure 2h shows the Seebeck coefficient of p‐type pastes depending on Bi_com_ powder contents. The obtained results show an increasing trend in the Seebeck coefficient of the Bi_com_ electrode at higher powder concentration conditions. This work also obtains high Seebeck coefficients (Figure 1c, over 100 μV K^−1^) comparable to that of the bulk Bi_com_^[^ ^50^ ^]^ for electrodes deposited from pastes with powder concentration conditions over 90%. This figure also shows photographs of the pastes under each powder concentration condition. The obtained photographs visually show powdery trends of the paste at higher Bi_com_ contents (solvent (black) and Bi_com_ (gray) in Figure 2a). Despite the higher Seebeck coefficients, such powdery trends degrade the conductivity of the pastes and electrodes. This situation is potentially because of lacking binder agents that facilitate electrical contacts between powder particles^[^ ^51^ ^]^ in pastes. In addition, degradations of the electrical conductivity hinder sensitive photodetection operations, as more significant thermal noise signals in controlling PTE sensors.^[^ ^52^ ^]^

To address these crucial issues, this work includes the surfactant as a part of the paste materials, as indicated earlier. Figure 2i shows a change in the electrical resistance values of Bi_com_ pastes against the surfactant content in them. Here, the Bi_com_ powder content is 90% for the above evaluation. The obtained result demonstrates a reduction in the electrical resistance of Bi_com_ paste in the surfactant content ranging up to 6 wt%. This situation infers that the surfactant relaxes the nonuniformity of electrical contacts between powder particles in Bi_com_ pastes. However, the electrical resistance of Bi_com_ pastes increases in the range of surfactant contents over 6 wt%. This trend is simply because the surfactant is electrically insulating.^[^ ^53^ ^]^ In addition to these improvements in electrical conductivity, Figure S5, Supporting Information, further improves the mechanical strength of Bi_com_ pastes using the surfactant. Figure 2j shows a change in the electrical resistance values of Bi_com_ electrodes against thermocompression temperature conditions after paste coating. Following the employment of the above surfactant, this work also performs the thermocompression process as a post‐treatment of Bi_com_ paste coating. The obtained results demonstrate reductions in the electrical resistance of Bi_com_ pastes by the thermocompression. The thermocompression process is as follows: putting the lamination film on the paste, setting the sample on the equipment, and hot pressing. This experimental situation suggests that the compression mechanically enhances contacts between Bi_com_ particles and that heating facilitates adhesive bonding of pastes by melted lamination films. Figure S6, Supporting Information, shows related experimental details of the thermocompression process. Regarding the coating consistency and accuracy of Bi_com_ pastes, Figure S7 and S8, Supporting Information, evaluates the shape and thickness properties of screen‐deposited films. Here, the existence of mask windows regulates the liquid‐like paste flowability within designated areas, maintaining sizes and shapes of screen‐coated thin‐films as designed in advance. Specifically, Figure S7, Supporting Information, demonstrates that the presenting screen‐coating method with laser‐processed window masks collectively suppresses size error ratios in Bi_com_ paste formations within 1.64% for three different shapes (square for 0.369 and 0.737%, triangle for 0.731 and 1.64%, and circle for 0.183%). For the remaining factor of film thicknesses in Bi_com_ paste coating, the aforementioned thermocompression approach offers error ratios within 5.59% across a 30 mm‐length channel with six scan spots (Figure S8, Supporting Information). Due to these supplementary evaluations, this work collectively satisfies fundamental TE conversion characteristics and operational high usability in handling Bi_com_ pastes as screen‐coatable material sources.

Following the design of these p‐type Bi_com_ materials, Figure 2k handles n‐type TE conversion pastes. The n‐type paste comprises the following materials: Bi_2_Te_3_ + Ru powder, surfactant, and CNT dispersion as the conductive solvent (including hydroxide and crown ether for electron doping). The TE conversion properties of the paste against powder contents show high n‐type Seebeck coefficients (<−100 μV K^−1^) at conditions of over 90%. For changing trends in the Seebeck coefficients of Bi_com_ pastes, Figure S9, Supporting Information, briefly describes theoretical estimations. As a numerical model indicates that the electrical conductivity of respective constituents potentially affects the total Seebeck coefficient of composite materials, the n‐type Bi_com_ paste employs CNT solvents with lower resistance values than that for the p‐types in this work. For the solvent in p‐type Bi_com_ pastes, “Device Materials” in Section 4 introduces detailed specifications. Based on the numerical model shown in Figure S9, Supporting Information, the CNT solvent dominantly governs the composite Seebeck coefficients of n‐type pastes up to a powder content of 70%, while the Bi_com_ agent plays a leading role in p‐type channels from lower concentration ranges. In summary, these interrelationships between respective constituents and the associated composite Seebeck coefficients of channels potentially guide specific strategies in designing further content materials in the presenting Bi_com_ pastes. By incorporating these findings and techniques, this work demonstrates freely coatable paste designs of high‐performance TE conversion materials for employing the all‐solution‐processable hybrid PTE sensor device structures.

### 2.3 Fundamental Device Performances of the All‐Paste‐Processable Hybrid PTE Sensor Sheet

**Figure 3:**
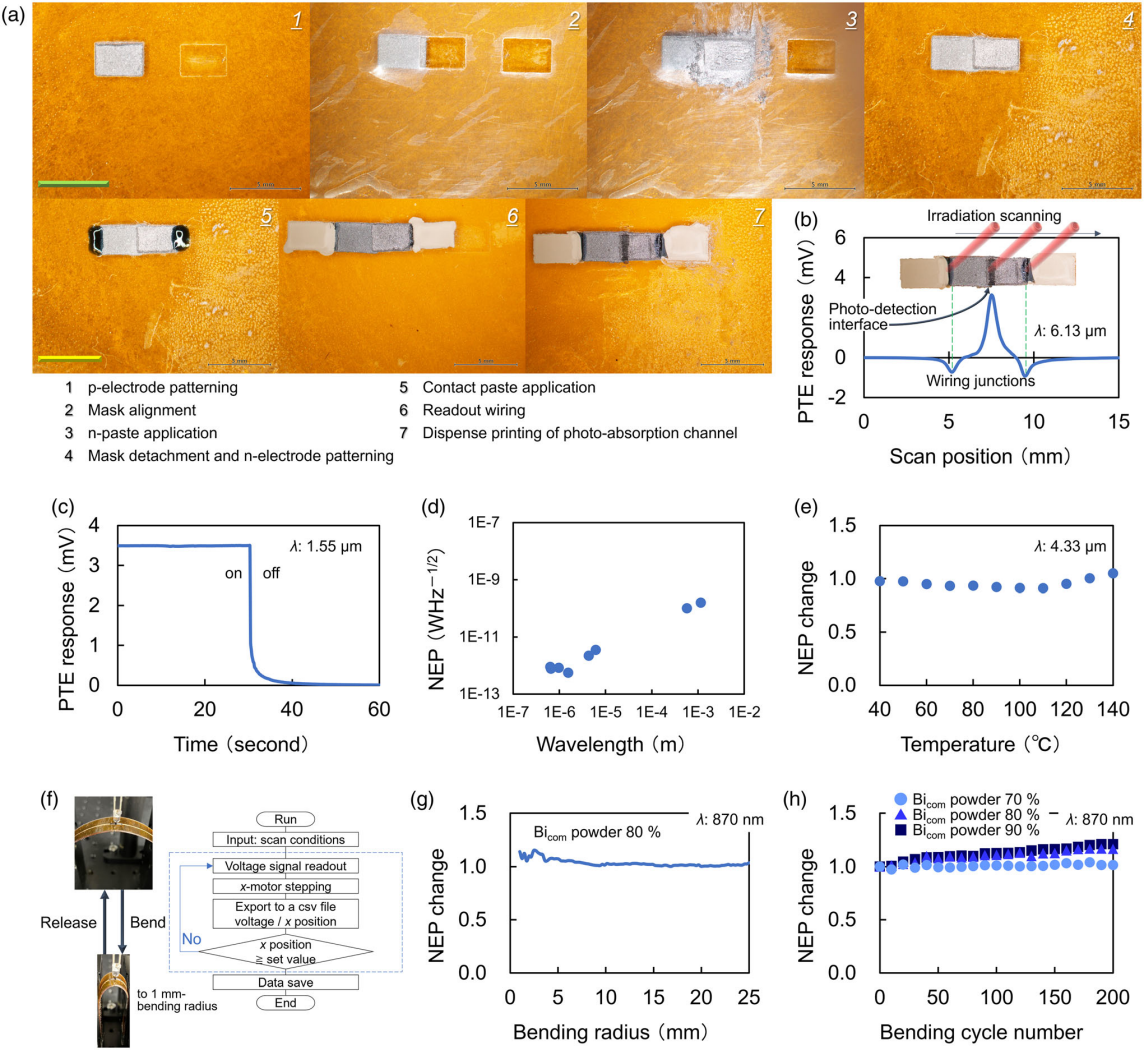
Fundamental optical performances of CNT film–Bi_com_ hybrid PTE sensors. a) Fabrication process of PTE devices. b) PTE response mapping by scanning laser irradiation in the device length direction. c) Transient response of the PTE device to external photoirradiation. d) Distribution of NEP values in ultrabroadband operations of the PTE device. e) Stability in photodetection sensitivities of the PTE device against the temperature change. f–h) Mechanical durability of the hybrid PTE sensor under external photoirradiation during bending deformations: experimental setups (f), NEP stability (g), and cyclic evaluations (h).

This work fabricates the sensitive hybrid PTE sensors by synergistically combining paste‐type high‐performance TE conversion electrodes with efficient photoabsorbent channel inks in a simple desktop all‐solution‐processable method. **Figure** **3**a shows the fabrication process of PTE devices. The specific process flow is as follows with numbering shown in the figure: 1 for p‐electrode patterning, 2 for mask alignment, 3 for n‐paste application, 4 for mask detachment and n‐electrode patterning, 5 for contact paste application, 6 for readout wiring, and 7 for dispense printing of photoabsorption channel. For the process numbering in the figure, “1” follows the screen‐coating method in Figure 2d–f, “2” aligns the overlap to 0.5 mm under optical microscope views, “3” and “4” are also available by screen coating, and “5” and “6” are preparatory steps toward connecting the device to the measuring equipment. This work employs a conductive silver particles–acrylic resin mixed paste for wiring as the all‐solution‐processable device fabrications. “5” further functions for the electrical and mechanical stability of the wiring. Finally, “7” completes the fabrication of the hybrid PTE device by printing CNT inks on the p–n Bi_com_ electrode junctions. In addition to explanations with “Device Fabrication” in Section 4, Figure S10, Supporting Information, introduces detailed experimental conditions in a flowchart manner regarding the above consecutive processes.

Figure 3b shows a PTE response mapping by scanning photoirradiation in the device length direction. The device shows the highest PTE response on the p–n junction across its structure. This work employs the p–n junction as the photodetection interface of the device. These results follow the effective Seebeck coefficient at each interface (p‐electrode: 116 μV K^−1^, n‐electrode: −86 μV K^−1^, and wiring: 1.5 μV K^−1^). Furthermore, the CNT film (photoabsorbing and heating channel) facilitates the ultrabroadband operations of the device, as shown in Figure 1d. Figure S11, Supporting Information, shows that the hybrid sensor structure in this work functions as the PTE effect device. Regarding these behaviors, the device performs photodetection operations at a speed of 154 ms (Figure 3c). This performance is comparable with the operating speeds of typical room temperature broadband sensors.^[^ ^54^, ^55^ ^]^ As an overview of the fundamental device performances, Figure 3d shows the photodetection sensitivities of the hybrid PTE sensor in ultrabroadband operations. This work employs noise equivalent power (NEP) values as a photodetection sensitivity index. NEP values are the ratio of noise voltage signals to normalized photoresponse intensities. The obtained results show that the proposed device in this work performs ultrabroadband sensitive photodetection operations (NEP values within 100 pWHz^−1/2^) in an uncooled and nonvacuum manner. In particular, the hybrid PTE sensor performs photodetection operations with a minimum NEP value of 560 fWHz^−1/2^. Furthermore, the photodetection wavelength bands of the presenting device exceed 1 μm–1 mm ranges. These performances emphasize that photodetection operations with the presenting hybrid PTE sensor are available in ultrabroadband regions over representative uncooled devices with comparable sensitivities to them^[^ ^56^, ^57^, ^58^, ^59^, ^60^, ^61^, ^62^ ^]^ (also see Figure S12, Supporting Information, for benchmarking). Here, Table S1 and S2, Supporting Information, summarize the fundamental device performances for typical photosensors. From another viewpoint, Figure S13, Supporting Information, simulates photodetection response signal intensities and the associated NEP values of the presenting hybrid PTE sensor regarding the powder content for Bi_com_ electrodes.

These photodetection performances of the hybrid PTE sensor also stably function at high operation temperature conditions (Figure 3e). This work evaluates a change in NEP values with the hybrid PTE sensor for operating temperature conditions. The obtained result suppresses error ratios of NEP values within 9% for high‐temperature operating conditions with the device over 100 °C. This situation is because the photoabsorption at the CNT film channel generates a local temperature increment even under high‐temperature conditions across the entire device structure.^[^ ^63^, ^64^ ^]^ In other words, the p–n junction interface with the photoabsorption channel exhibits the largest thermal gradient owing to local heating in addition to high operation temperatures (reference value) across the device. Therefore, photoinduced temperature gradients across the device, associated PTE response signals (Figure S14, Supporting Information), and subsequent NEP values are stable for changes in the reference device operating temperature, as indicated earlier. Following these fundamental behaviors, Figure S15 and S16, Supporting Information, presents further device specifications of the hybrid sensor to facilitate a deeper understanding of PTE designs: responses against photoirradiation with different intensities and thermal monitoring. In addition to the above physical robustness and stability, Figure 3f–h and Figure S17, Supporting Information, also evaluate the mechanical durability of the hybrid PTE sensor. The presenting device stably maintains its inherent photodetection sensitivities under repetitive bending configurations over 200 cycles of the whole structure within 20% NEP value changes for the 90% Bi_com_ powder content condition in TE electrodes. This work even clarifies that the management of powder contents further enriches such physical durabilities of the presenting hybrid PTE sensor structure as follows: photodetection operations within 15 and 5% NEP value changes for 80, and 70% Bi_com_ amount conditions in TE electrodes. These evaluations (fundamental operation, stability, and durability) demonstrate that the hybrid PTE sensors proposed in this work facilitate diverse sensing applications in both optical and physical fields.

**Figure 4:**
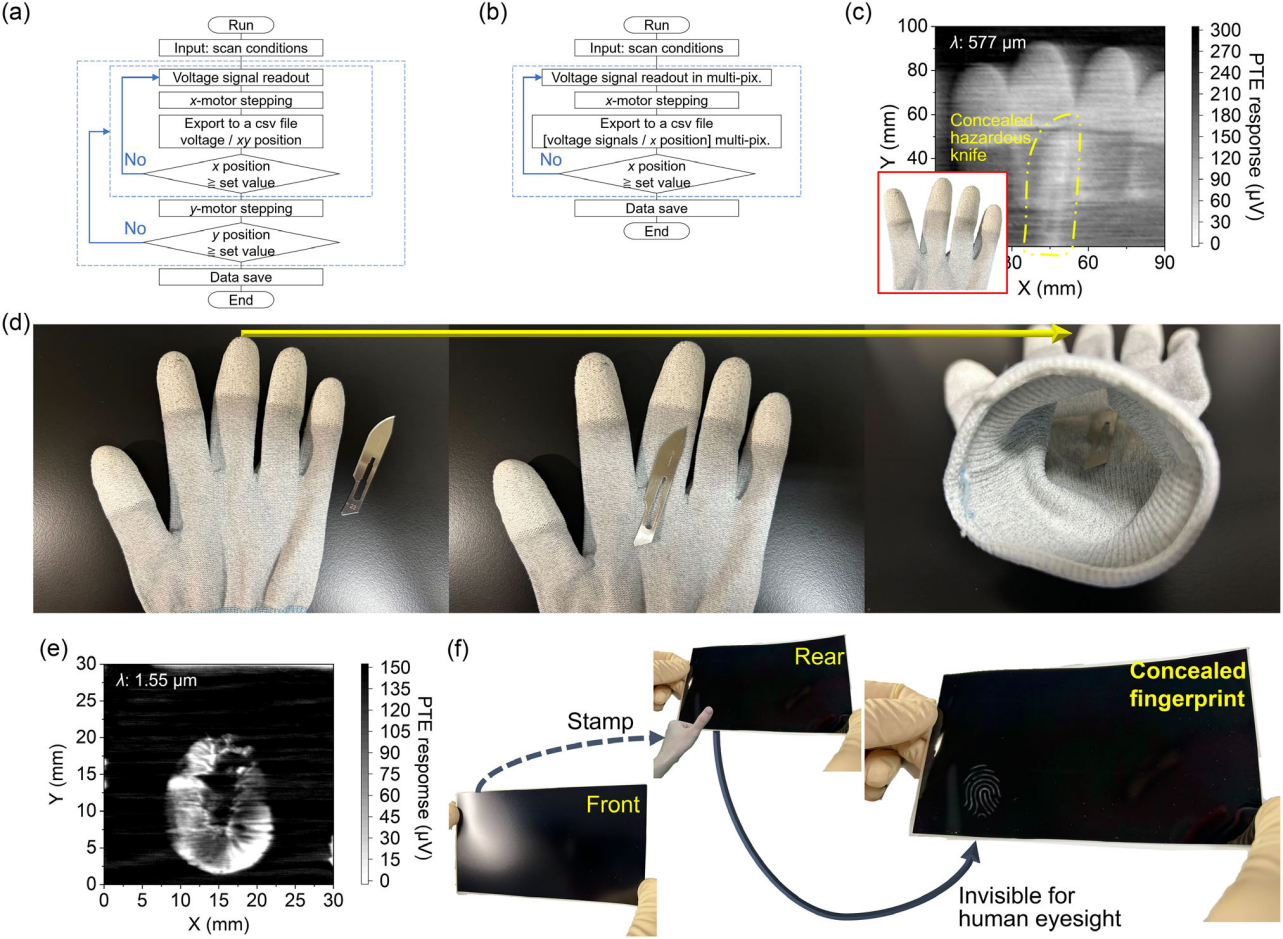
Longer‐wavelength photoimaging with the hybrid PTE sensor. a,b) Flowcharts of experimental setups with a single‐pixel (a) and multiple‐integrated (b) sensors. c) Transmissive *xy*‐scan THz imaging with the hybrid PTE sensor of the hazardous defective glove. d) Target preparation in (c). e) Transmissive *xy*‐scan IR imaging with the hybrid PTE sensor of the opaque sheet. f) Target preparation in (e).

With the aforementioned photodetection behaviors, the use of the hybrid PTE sensor also results in appropriate operations for optical imaging. As an example of the above concept, this work first demonstrates nondestructive imaging with the hybrid PTE sensor. To prepare experimental setups for imaging measurements, **Figure** **4**a,b first introduces data processing flowcharts that synchronize the hybrid PTE sensor, photosources, a data logger, digital stepping motor stages, and monitoring targets within a single‐system packaging. “Signal readout” in Section 4 further describes respective system constituents. Based on this experimental setup, Figure 4c shows the result of transmissive 2D‐scan THz imaging with a single‐pixel hybrid PTE sensor device. Here, the target of this measurement is an experimental protective glove. The target contains a concealed hazardous knife on its inside (Figure 4d). This hazardous knife is indistinguishable from the outside of the glove with human eyes. This situation potentially causes injury to the user. For the above sample situation, the obtained result clearly visualizes the concealed hazardous knife inside the glove. In the 2D PTE image, the darker and brighter color scale corresponds to the transmissive response intensities of the hybrid PTE sensor. Based on these preparations, the device comprehensively detects local reductions in transmission THz signals due to surface photoreflections at the knife part (brighter than surroundings), clearly visualizing thicker fingertips (stronger absorption) of the target as well. Here, this work fixes the positions of the hybrid PTE sensor and the THz source and scans the spatial coordinates of the target. In other words, the horizontal and vertical axes of the obtained PTE image respectively correspond to *xy* scanning directions of the target in a 2D spatial coordinate. The inherent permeability of THz irradiation to nonmetallic materials and the associated sensitive operation of the hybrid PTE sensor contribute to the presenting nondestructive testing demonstration.

In addition to this THz imaging, the advantageous optical properties of the hybrid PTE sensor also facilitate shorter wavelength and high‐resolution IR nondestructive testing. Figure 4e then handles a fingerprint hidden behind a black opaque sheet in nondestructive IR imaging with the hybrid PTE sensor. This work performed finger stamping with a black ink on the rear side of the above sheet (Figure 4f). This situation means that the fingerprints are indistinguishable from the front sheet side with human eyes. Here, the hybrid PTE sensor collectively detects IR irradiation (penetrating the opaque sheet and local reductions in transmission signals due to absorption phenomena in the fingerprint area). Together with the aforementioned device operations and the associated performances, the presenting material and structural strategies in designing the hybrid PTE sensor lead to demonstrating such fundamental longer‐wavelength photomonitoring.

### 2.4 Functional Nondestructive Inspections with the Hybrid PTE Sensors

**Figure 5:**
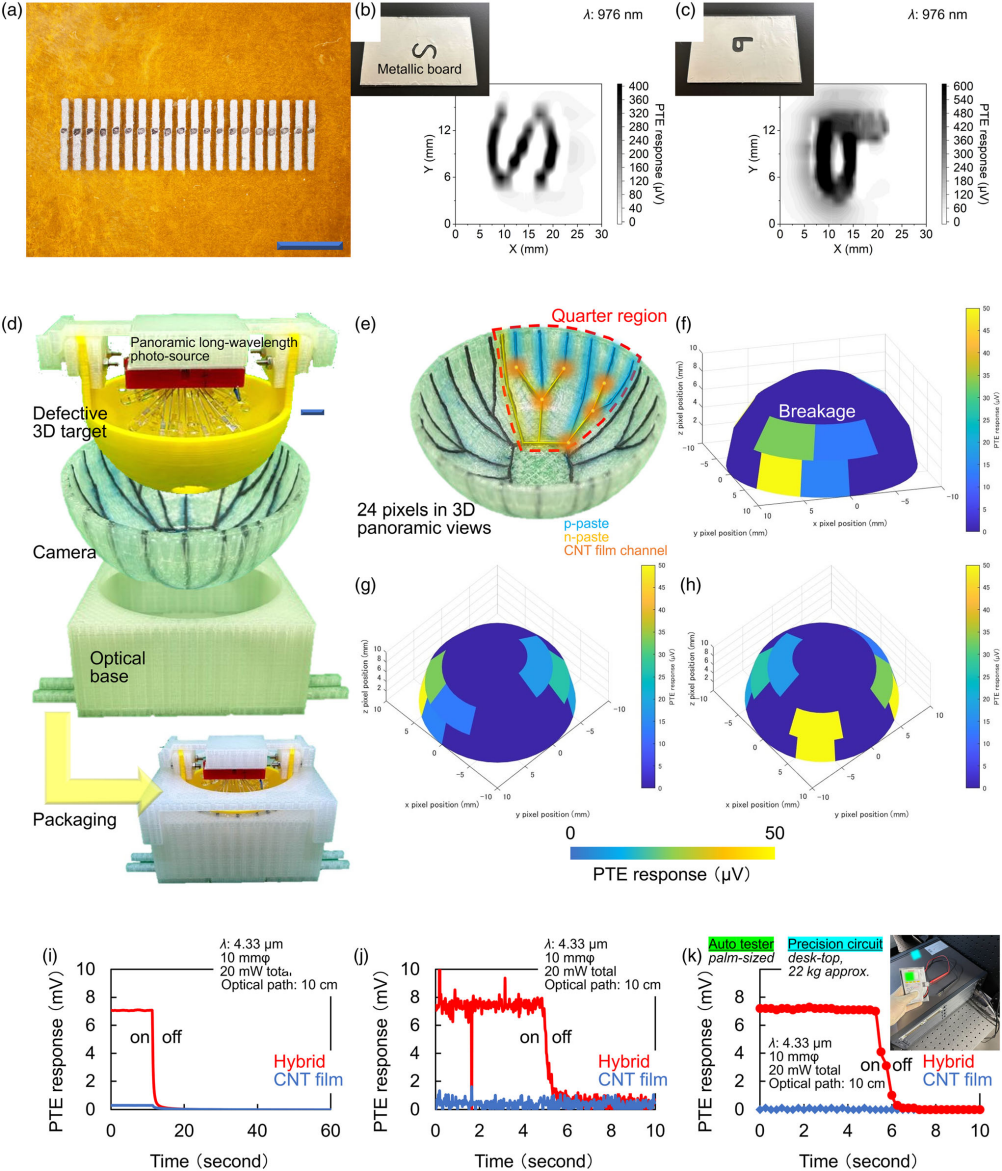
Functional monitoring applications with hybrid PTE sensors. a) Photograph of the multipixel integrated sensor array device. b,c) Imaging measurements with the hybrid PTE sensor array device for 10 pixels (b) and 20 pixels (c) integrated structures. d) Experimental setup for 3D imaging with the bowl camera. e) Photograph of the hybrid PTE bowl camera. f–h) Nondestructive 3D panoramic visualization testing for a defective bowl object in side‐, front‐, and rear‐bird's eye views (f: side, g: front, h: rear). i,j) IR detecting operations with PTE sensor devices for each signal readout equipment (via i: bulky high precision analog circuit and j: typical portable oscilloscope). k) Device operation of PTE sensors with a portable voltage tester in detecting external photoirradiation.

Based on the fundamental characterization of the hybrid PTE sensor, this work finally demonstrates functional monitoring applications. **Figure** **5**a–c first demonstrates integrations of hybrid PTE sensors and applications as imaging devices. In general, the integration of image sensor pixels shortens the measurement time.^[^ ^65^ ^]^ For example, the configurations for imaging measurements with integrated styles of sensors are as follows: single pixel for *xy*‐scanning, 1D array for uniaxial moving, and 2D camera for real‐time operations. Here, this work designs a 1D array structure, as shown in Figure 5a. Figure 5b,c next demonstrates imaging measurements with the hybrid PTE sensor array device for 10 pixels (b)‐ and 20 pixels (c)‐integrated structures. The pixel pitches of the utilized sensor array devices are 2 mm (Figure 5b) and 1 mm (Figure 5c). The fabrication process of the sensor array devices is as follows: preparing masks with aligned windows for p‐/n‐type Bi_com_ pastes, respectively, coating them alternately for the p–n junction formation, and applying each CNT film photothermal channel. Figure S18, Supporting Information, shows specific experimental setups for this measurement. This work obtains 2D PTE images by uniaxial scanning in transmission systems. This measurement also conceals metallic lettering patterns behind an opaque plate as imaging targets. In particular, this work shaves metal plates in accordance with alphabets “S” and “P”. Here, the hybrid PTE sensor array device sensitively detects penetrated longer‐wavelength photoirradiation through the object. As shown in the obtained PTE images, the sensor array device correctly visualizes the object with only uniaxial scanning in a nondestructive manner. In other words, the sensor array device detects locally transmitted photoirradiation consistent with the shape of lettering patterns. Based on the above results, the hybrid PTE sensors comprising all‐solution‐processable materials are suitable for multiple‐pixel integrations. Here, Figure S19, Supporting Information, briefly describes a signal calibration process for these transmissive PTE imaging measurements with the presenting hybrid sensor array device. In addition, Figure S20, Supporting Information, introduces a preparation flow of the imaging target in Figure 5a–c. At this moment, spatial resolutions in photoimaging by this work are 1 mm for the pixel pitch of the integrated hybrid PTE sensor array and 1 mm for uniaxial stepped scanning with optical stages (perpendicular to the device alignment) in Figure 5c, for example. As mentioned earlier, the experimentally obtained minimum line and space value (256 μm: Figure S4, Supporting Information) in screen coating of Bi_com_ pastes infers that upcoming optimizations in material forming conditions (e.g., adhesion and mask thicknesses) potentially develop integrated PTE devices with higher spatial resolutions. For single‐pixel operations of the hybrid PTE sensor (e.g., Figure 4c,e), this work sets a stepping length in stage *xy*‐scanning (spatial resolution) of 250 μm.

Sensitive photodetection performances and the all‐solution‐processable device fabrication process of these hybrid PTE sensors further facilitate 3D imaging applications. In particular, constituent materials of the hybrid PTE sensor are freely paintable as liquid pastes, regardless of the size and shape of substrates. As an example of such conceptualizations, Figure 5d demonstrates the curvilinear integration of hybrid PTE sensors as a bowl camera and nondestructive 3D panoramic visualization testing. This work first paints pastes constituting the hybrid PTE sensor on the inner wall of the bowl (Figure S21, Supporting Information). Figure 5e shows a photograph of the hybrid PTE bowl camera fabricated in this work. The constituent pastes of the hybrid PTE sensor are adaptable not only to mechanical screen coating (as shown in Figure 3) but also to flexibly designable manual painting. In other words, this work performs large‐area integrations of the hybrid PTE sensors with high yields by mechanical screen coating on flat substrates and manual painting on the 3D object. Based on these device preparations, this demonstration handles a bowl‐shaped defective object. This object contains several breakages in its curvilinear structure. As shown in Figure S22, Supporting Information, the use of conventional flat‐view cameras is insufficient for comprehensive monitoring of all defects in a single measurement due to 3D blind spots. For the earlier situation, this work flexibly designs the bowl camera according to the structure and size of the object and demonstrates omnidirectional imaging in a transmissive system combined with a panoramic longer wavelength source (Figure S23, Supporting Information) without blind spots. Based on these experimental setups, Figure 5f–h demonstrates nondestructive 3D panoramic visualization testing for the defective bowl object in side‐, front‐, and rear‐bird's eye views (Figure 5f: side, Figure 5g: front, Figure 5h: rear). The obtained 3D PTE images comprehensively visualize each defect of the object within a single signal‐readout measurement without spatial scanning. In these 3D PTE images, each pixel of the bowl camera detects local photoirradiation penetrating the defects. Here, this work prepared the presenting 4D diagram (*xyz*‐coordinate and PTE‐based color scale) by referring to spatial positions and photoresponses of each pixel in the bowl camera. In these demonstrations, “3D printing” in Section 4 briefly describes details for modeling processes of the target and substrate.

In addition to such handling of materials and devices in an all‐solution‐processable manner, the hybrid PTE sensor proposed in this work further improves its usability. As the above conceptualization, this work focuses on the coupling of PTE devices and the signal readout equipment. In particular, Figure 5i,j demonstrates fundamental IR sensing by two PTE devices with different signal readout equipment. Here, this work prepares a typical unseparated CNT film‐based sensor (known as one of the representative PTE devices) for benchmarking with the presenting hybrid design. As described earlier, the CNT film, which serves as the photothermal channel material of the hybrid structure in this work, also functions as a PTE sensor by itself.^[^ ^66^ ^]^ For these measurements, the employed signal readout equipment is as follows: a bulky high‐precision analog circuit (Figure 5i) and a typical portable oscilloscope (Figure 5j). The obtained result first demonstrates appropriate IR sensing by both PTE devices with the bulky high‐precision analog circuit, as shown in Figure 5i. Whereas, the typical portable oscilloscope selectively handles appropriate IR sensing signals with the hybrid PTE device. Here, the Seebeck coefficient of Bi_com_ pastes (playing a leading role in this work) facilitates such advantageous behaviors of the hybrid PTE sensor described earlier. The effective Seebeck coefficient of Bi_com_ pastes exceeding 240 μV/K provides PTE response signals with the hybrid sensor ≈7 mV for MIR irradiation (*λ:* 4.33 μm, 10 mmφ, 20 mW total in a beamspot, and optical path length: 10 cm). In contrast, the CNT film PTE sensor reduces its response signal intensity to ≈300 μV for the above MIR irradiation due to the Seebeck coefficient of ≈40 μV K^−1^ with corresponding constituent materials. Conventional PTE device designs mainly focus on improving operation sensitivities through suppressing noise signals.^[^ ^21^, ^22^ ^]^ Here, the representative PTE device materials (e.g., unseparated CNT films, graphene, and PEDOT: PSS) typically exhibit the Seebeck coefficients of a few tens of μV K^−1^,^[^ ^27^, ^28^, ^29^, ^30^, ^31^ ^]^ resulting in hundreds of μV response signals as sensors against external photoirradiation.^[^ ^23^, ^24^ ^]^ Nevertheless, photodetection operations of PTE sensor devices with a higher‐response voltage signal range are essential, together with recent developments and their potential suitability for nondestructive testing applications. This situation is mainly because the signal readout equipment in more compact and portable setups generally regulates detectable voltage signals to higher ranges than bulky types.^[^ ^25^, ^26^ ^]^ In other words, handling lower‐range responses of PTE sensor devices requires bulky configurations of the signal readout equipment (for high‐precision performances). This situation potentially hinders opportunities for utilizing PTE sensors as portable nondestructive testing devices in onsite environments. Regarding these, Figure 5k shows photographs of two different signal readout equipment. Their readout resolutions are as follows: 100 nV for the high‐precision circuit and 0.1 mV for the digital tester. Based on the earlier experimental setup, the obtained result shown as transient waveforms and Figure S24 and S25, Supporting Information, demonstrates fundamental IR sensing by two PTE devices with the palm‐sized digital tester. As shown, the hybrid PTE sensor proposed in this work is fully controllable with the portable signal readout equipment. Through these series of functional monitoring demonstrations, all‐solution‐processable sensor device designs combined with Bi_com_ paste electrodes and CNT film photothermal channels facilitate user‐friendly operations of PTE measurements.

## 3 Conclusion

Based on these experimental demonstrations, the hybrid PTE sensors proposed in this work advantageously facilitate nondestructive testing with high usability among existing electronic devices. Specifically, **Table** **1** compares the representative user‐friendly paste‐type sensor devices reported in recent years.^[^ ^67^, ^68^, ^69^, ^70^, ^71^, ^72^ ^]^ Remarkable achievements in research fields of liquid materials enable the paste treatment of various sensor devices. Here, the representative paste materials and sensor types are as follows: nanocarbon, metal nanowire, liquid metal, and perovskite for inks, electrochemical, strain, photodetectors, photoluminescent, solar cells, and thermistors for sensors. Among those previous works, the proposed hybrid PTE sensor realizes ultrabroad MMW–vis band photomeasurements and functional nondestructive testing applications while maintaining usability comparable to existing paste devices. Furthermore, these paste sensor devices are easily formable and potentially function as complementary multimodal systems through upcoming combinatorial fabrications.

**Table 1:** Comparison of the fundamental features for the representative paste‐like sensors.

Sensor type	Material	Processing accuracy	Target substrate	Application
This work	CNT ink, Bi_com_ paste	256 μm	On universal substrates (thin‐soft, 2D, 3D, rigid)	Nondestructive imaging
Electrochemical^[^ ^67^ ^]^	Polyfunctionalized graphite	N.A.	Ceramic	Vanillin monitoring in foods
Strain^[^ ^68^ ^]^	Carbon black, silver paste	100 μm	On designated polymer sheets	Wearable motion monitoring
PD^[^ ^69^ ^]^ a)	Meshed Si, silver paste	N.A.	Self‐standing	Bendable display
PL^[^ ^70^ ^]^ a)	Liquid metal (Ga, Bi, In, Sn)a)	20 mm	Ceramic	Fluorescent display
PV^[^ ^71^ ^]^ a)	Perovskite	N.A.	FTOa) glass	Energy harvesting
Temperature^[^ ^72^ ^]^	BFOa)	500 μm	FR‐4a)	Thermistor

a) PD, photodiode; PL, photoluminescence, PV, photovoltaic; Si, silicon; Ga, gallium; In, indium; Sn, tin; BFO, bismuth ferrite; FTO, fluorine‐doped tin oxide; FR‐4, thermally boosted glass‐reinforced epoxy flame‐retardant four.

In conclusion, the presenting hybrid PTE sensor detects external photoirradiation with a minimum NEP value of 560 fWHz^−1/2^ in an operable wavelength band of 660 nm–1.15 mm, assisted by the advantageous effective Seebeck coefficients over 240 μV K^−1^. In addition, the proposed devices stably function as photosensors with a fabrication size accuracy of 256 μm at minimum, in a wide temperature range over 100 °C (NEP error: within 9%), and even under repetitive mechanical deformations (NEP error: within 5–20% against 200‐cyclic bending). Owing to these fundamentals, the sensitive, ultrabroadband, and robust paste‐like sensors are fully available in a diverse user‐friendly manner (e.g., large‐area multiple‐pixel integrations, 3D panoramic formations for around‐view photomonitoring without blind spots, and portable signal‐control setups). Following these, further related efforts potentially enrich the functionality of the hybrid PTE sensor. One specific example is controlling particle sizes of Bi_com_ powders,^[^ ^73^ ^]^ which expands device fabrication methods to air‐jet type dispense printing in addition to the proposed screen coating and manual painting. From another viewpoint, device design strategies of stretchable PTE pastes by mixing acrylic elastomers^[^ ^74^ ^]^ also play an essential role in developing further multimodal wearable Bi_com_–CNT‐based sensitive and high‐usability photo‐monitoring modules, for example.

## 4 Experimental Section

4.1

4.1.1

##### Device Materials

For device fabrication, this work employed the following materials: p‐type bismuth composite chip/powder (Bi_0.3_Sb_1.7_Te_3_, Toshima Manufacturing Co., Ltd.), n‐type bismuth composite chip/powder (Bi_2_Te_3_ + Ru, Toshima Manufacturing Co., Ltd.), photoabsorbent CNT channel (EC‐DH, Meijo Nano Carbon Co., Ltd), p‐type solvent (poly(3,4‐ethylenedioxythiophene)‐poly(styrenesulfonate) 739316‐25G, Sigma‐Aldrich Co. LLC), and n‐type solvent. Here, the n‐type solvent consisted of CNT solutions (ZEONANO SG101, Zeon Co.), 15‐crown 5‐ether dispersions (C0859, Tokyo Chemical Industry Co. Ltd.), and potassium hydroxide (0.5 m KOH, Tokyo Chemical Industry Co. Ltd.). In the above materials, CNTs are single‐walled semiconducting‐metallic‐mixed types for the photoabsorbent channel and solvent. The remaining composition included readout wiring (ELEPASTE NP1, TAIYO INK MFG Co. Ltd.), contacting pastes (poly(3,4‐ethylenedioxythiophene)‐poly(styrenesulfonate) (768650‐25G, Sigma‐Aldrich Co. LLC)), and surfactants (ESLEAM AD‐3172, NOF Co.).

##### Seebeck Coefficient Measurement

For measuring the Seebeck coefficient of device materials, this work used microceramic heaters (MS‐M1000, SAKAGUCHI E.H. VOC Co.), K‐type thermocouples (T‐35K, SAKAGUCHI E.H. VOC Co.), and a digital multiplexer (DAQ970A, KEYSIGHT TECHNOLOGIES Inc.). The system recorded the TE voltage responses generated by a 5 °C temperature difference between the heaters via the K‐type thermocouple probed at both ends of device materials. The K‐type thermocouple consisted of two terminals (alumel: −18μV K^−1^ and chromel: 22 μV K^−1^).^[^ ^63^ ^]^

##### Spectroscopy

This work performed spectroscopy measurements in three different bands. In vis–NIR bands, the employed spectrometer was “UV–vis–NIR” (UV‐2600i, ISR‐2600Plus, Shimazu Co.). In MIR–FIR bands, the employed spectrometer was “FTIR” (IRTracer‐100, Shimadzu Co.). In THz–MMW bands, the employed spectrometer was “THz‐TDS” (TAS7 × 00TS, Advantest Co.). The related previous works^[^ ^46^, ^64^ ^]^ further specified detailed experimental conditions for these spectroscopy units.

##### TE and PTE Effect

In operating the CNT sensor, the following equation briefly describes its signal against external electromagnetic‐wave/photoirradiation

(1) $$\Delta V=S_{\text{eff}}\times \Delta T=\left(S_{p}-S_{n}\right)\times \Delta T$$

where Δ*V*, *S* (_eff_: effective and _p/n_: p‐type‐/n‐type‐films), and Δ*T*, respectively, correspond to the direct‐current (DC) voltage response by the device, the Seebeck coefficient of constituent materials, and the absorption‐induced temperature gradient across the channel.^[^ ^17^ ^]^ The above situation represents that operations of CNT film PTE sensors synergized typical TE conversion steps (right‐hand side of Equation (1), temperature gradient in a contact manner) and noncontact photoabsorption‐induced heating. Based on the above, the following equation briefly describes the operation model of the presenting hybrid PTE sensor device

(2) $$\Delta V=S_{\text{eff}}\times \Delta T=\left(S_{p‐\text{Bi}}-S_{n‐\text{Bi}}\right)\times \Delta T$$

where *S*_p‐Bi_ and *S*_n‐Bi_, respectively, correspond to the Seebeck coefficients of the p‐/n‐type Bi_com_ pastes.

##### Signal Readout

To read out DC voltage PTE responses of the device, this work employed a multiplexer data logger (34980A‐34923A/T, KEYSIGHT TECHNOLOGIES Inc.). The scan resolution of the data logger was 100 nV at a maximum readout speed of 6.6 Hz. There can be up to 80 elements for a terminal block of the data logger. Furthermore, the data logger controlled up to eight terminal blocks. The device had two (ground and readout) wiring terminals connected to the data logger and then transferred and recorded DC‐voltage PTE responses to LabVIEW programs in a PC via a General Purpose Interface Bus (GPIB) cable. The portable oscilloscope and digital tester employed in this work were as follows: PM3 (Sanwa Electric Instrument Co., Ltd.) and View Go (IWATSU ELECTRIC Co., Ltd.). For spatial scanning or imaging measurements with the device, this work employed a digital stepping motor stage (MORTARIZED STAGE, SIGMAKOKI Co.) and a stage controller (SHOT‐304GS, Sigma Koki Co.). The minimum stepping resolution was 500 nm in *xyz* directions.

##### Photosources

The nine types of photosources employed in this work were as follows: an emitter (10 MHz–24 GHz) dual‐channel microwave generator, (SynthHD PRO (v2), Windfreak Technologies LLC), broadband horn antenna (4.5–50 GHz 13 dB Gain 2.4 mm female, LB‐45 500), −2.4 F, A‐INFO Inc. Ltd., ultrawide‐band power amplifier (0.04–30 GHz, R00G30GSPG, RF‐Lambda LLC), and a frequency multiplier (*λ* = 1.15 mm, Custom Modular Tx‐Transmitter, Virginia Diodes Inc.) in MMW bands, a customized frequency multiplier (*λ* = 577 μm, 520‐ to 532‐GHz Custom Modular Tx‐Transmitter, Virginia Diodes Inc.) in a THz band, quantum cascade lasers (*λ* = 6.13 μm: L12006‐1631 H‐E and *λ* = 4.33 μm: L12004‐2310 H‐E, Hamamatsu Photonics K.K.), semiconducting laser fiber diodes (*λ* = 1.55 μm: FPL1009P and *λ* = 976 nm: BL976‐PAG900, Thorlabs, Inc.), light‐emitting diodes (*λ* = 870 nm, L12170, Hamamatsu Photonics K.K.) in IR bands, and a semiconducting laser fiber diode (*λ* = 660 nm, LP660‐SF50, Thorlabs, Inc.) in a vis band.

##### Device Fabrication

This work used a desktop CO_2_ laser processing system (HAJIME CL1, Oh‐Laser Co. Ltd.) for mask‐window processing. The processing conditions were 15 W irradiation in the wavelength of 10.6 μm at a speed of 30 cm s^−1^, and the minimum processing resolution was 300 μm. This work employed a mechanical coater (Smart desktop coater TC‐100s, Mitsui Electric Co. Ltd.) and a bar applicator (OSP‐1.5, Mitsui Electric Co. Ltd.) for screen coating of constituent materials of PTE devices.^[^ ^49^ ^]^ For the device fabrication process, this work also utilized 25 μm‐thick double‐sided adhesive PI films (NO.7601, TERAOKA SEISAKUSHO Co. Ltd.), coating masks: film separator of the PI film, 50–125 μm‐thick PI masks (Kapton, Dupont‐Toray Co.), and supporting quartz glass substrates (S9213, Matsunami Glass Ind., Ltd.). For printing the photoabsorbent CNT channel, this work employed a noncontact jet dispenser consisting of a desktop robot (SHOTMASTER300ΩX SM300OMEGAX‐3A‐SS, Musashi Engineering, Inc.), a control unit (Hyper Jet2 MJET‐H‐2‐CTR3, Musashi Engineering, Inc.), and a stage temperature controller (TCU‐02‐MU, Musashi Engineering, Inc.). The software for controlling the robot was 350PCSMART (Musashi Engineering, Inc.). This work also installed MuCADV software (Musashi Engineering, Inc.) for editing printing patterns. The equipment enabled ink dispensing at an air pressure of 600 kPa, and the nozzle moved at a speed ranging from 10 to 30 mm s^−1^ with a 250 mm square stage. The thermocompressor was OPL‐200‐10 (FUJIIMPULSE Co., Ltd.).

##### 3D Printing

For stereoscopic omnidirectional imaging, this work fabricated the bowl‐shaped supporting substrate using a 3D resin printer (Value 3D Magix MF‐2500 EP II, MUTOH INDUSTRIES Ltd., CAD software: AUTODESK Tinkercad). The minimum processing conditions were 50 μm in *xy* and 100 μm in *z* directions, and the maximum size (fabrication area) was 300 mm cube. Owing to the maximum temperature of the head/stage units (300 and 150 °C), the 3D resin printer utilized resins of polylactic acid and acrylonitrile–butadiene–styrene.

## Conflict of Interest

The authors declare no conflict of interest.

## Author Contributions

**Yuto Matsuzaki**: investigation (supporting); writing—original draft (lead). **Reiji Tadenuma**: investigation (lead). **Yuto Aoshima**: investigation (lead). **Minami Yamamoto**: investigation (supporting). **Leo Takai**: investigation (supporting). **Yukito Kon**: investigation (supporting). **Daiki Sakai**: investigation (supporting). **Norika Takahashi**: investigation (supporting). **Ryo Koshimizu**: investigation (supporting). **Qi Zhang**: investigation (supporting). **Naoko Hagiwara**: investigation (supporting). **Meiling Sun**: investigation (supporting). **Daiki Shikichi**: investigation (supporting). **Raito Ota**: investigation (supporting). **Sayaka Hirokawa**: investigation (supporting). **Yukio Kawano**: conceptualization (supporting); funding acquisition (equal); project administration (supporting); supervision (supporting). **Kou Li**: conceptualization (lead); data curation (lead); formal analysis (lead) funding acquisition (equal); investigation (supporting); methodology (lead); project administration (lead); resources (lead); supervision (lead); validation (lead); visualization (lead); writing—original draft (supporting); writing—review and editing (lead). **Yuto Matsuzaki**, **Reiji Tadenuma**, and **Yuto Aoshima** contributed equally to this work and co‐first authors.

## Supporting information

Supplementary Material

## Data Availability

The data that support the findings of this study are available from the corresponding author upon reasonable request.

## References

[1] K. Brinker , M. Dvorsky , M. T. A. Qaseer , R. Zoughi , Philos. Trans. R. Soc. A 2020, 378, 20190585. 10.1098/rsta.2019.0585 32921242

[2] S. L. Chen , C. Tian , Vis. Comput. Ind. Biomed. Art 2021, 4, 6. 33740149 10.1186/s42492-021-00073-1 PMC7979856

[3] S. S. Kim , S. K. Yong , W. Kim , S. Kang , H. W. Park , K. J. Yoon , D. S. Sheen , S. Lee , C. S. Hwang , Adv. Mater. 2023, 35, 2200659. 10.1002/adma.202200659 35305277

[4] M. Javaid , A. Haleem , S. Rab , R. P. Singh , R. Suman , Sens. Int. 2021, 2, 100121. 10.1016/j.sintl.2021.100117 PMC8590973 34806053

[5] M. A. Abou‐Khousa , M. S. U. Rahman , K. M. Donnell , M. T. A. Qaseer , IEEE Trans. Instrum. Meas. 2023, 72, 1.37323850

[6] C. Guo , W. Xu , M. Cai , S. Duan , J. Fu , X. Zhang , IEEE Access 2022, 10, 121547.

[7] G. Dua , V. Arora , R. Mulaveesala , IEEE Sens. J. 2021, 21, 7940.

[8] I. Goryanin , S. Karbainov , O. Shevelev , A. Tarakanov , K. Redpath , S. Vesnin , Y. Ivanov , Drug Discovery Today 2020, 25, 757. 32004473 10.1016/j.drudis.2020.01.016

[9] M. Martin , A. Chong , F. Biljecki , C. Miller , Renewable Sustainable Energy Rev. 2022, 165, 112540.

[10] Y. Jiang , G. Li , H. Ge , F. Wang , L. Li , X. Chen , M. Lu , Y. Zhang , IEEE Access 2022, 10, 53761.

[11] L. Legan , T. Leskovar , M. Črešnar , F. Cavalli , D. Innocenti , P. Ropret , J. Cult. Heritage 2020, 41, 13. 10.3390/ma18040742 PMC11857665 40004266

[12] M. Li , F. Igbari , Z. K. Wang , L. S. Liao , Adv. Energy Mater. 2020, 10, 2000641.

[13] M. J. M. Hosseini , R. A. Nawrocki , Micromachines 2021, 12, 655.34199683

[14] P. C. Y. Chow , T. Someya , Adv. Mater. 2020, 32, 1902045. 10.1002/adma.201902045 31373081

[15] L. Li , D. Wang , D. Zhang , W. Ran , Y. Yan , Z. Li , L. Wang , G. Shen , Adv. Funct. Mater. 2021, 31, 2104782.

[16] X. Lu , L. Sun , P. Jiang , X. Bao , Adv. Mater. 2019, 31, 1902044. 10.1002/adma.201902044 31483546

[17] K. Li , Y. Kinoshita , D. Sakai , Y. Kawano , Micromachines 2023, 14, 61. 10.3390/mi14010061 PMC9865119 36677122

[18] X. Xu , N. M. Gabor , J. S. Alden , A. M. van der Zande , P. L. McEuen , Nano Lett. 2010, 10, 562. 20038087 10.1021/nl903451y

[19] X. Cai , A. B. Sushkov , R. J. Suess , M. M. Jadidi , G. S. Jenkins , L. O. Nyakiti , R. L. Myers‐Ward , S. Li , J. Yan , D. K. Gaskill , T. E. Murphy , H. D. Drew , M. S. Fuhrer , Nat. Nanotechnol. 2014, 9, 814. 25194945 10.1038/nnano.2014.182

[20] M. Dai , C. Wang , B. Qiang , Y. Jin , M. Ye , F. Wang , F. Sun , X. Zhang , Y. Luo , Q. J. Wang , Nat. Commun. 2023, 14, 3421. 37296149 10.1038/s41467-023-39071-7 PMC10256712

[21] K. J. Erikson , X. He , A. A. Talin , B. Mills , R. H. Hauge , T. Iguchi , N. Fujimura , Y. Kawano , J. Kono , F. Léonard , ACS Nano 2015, 9, 11618. 26512738 10.1021/acsnano.5b06160

[22] M. Zhang , D. Ban , C. Xu , J. T. W. Yeow , ACS Nano 2019, 13, 13285. 31715095 10.1021/acsnano.9b06332

[23] J. Wang , Z. Xie , J. A. Liu , J. T. W. Yeow , J. Mater. Chem. C 2022, 10, 15105.

[24] M. Dai , C. Wang , B. Qiang , F. Wang , M. Ye , S. Han , Y. Luo , Q. J. Wang , Nat. Commun. 2022, 13, 4560. 35931776 10.1038/s41467-022-32309-w PMC9356042

[25] MSR Electronics GmbH , *Wireless Data Logger MSR145WD with Bluetooth and FlexSensors*, https://www.msr.ch/media/pdf/Wireless‐Data‐Logger‐MSR145WD‐BLE‐Datasheet‐EN.pdf (accessed: August, 2024).

[26] Sanwa Electric Instrument Co., Ltd ., *CD721 Digital Multimeter Instruction Manual*, https://www.sanwa‐meter.co.jp/japan/pdf/manual/digital_multimeters/CD721_JP.pdf?channel=sanwa (accessed: August, 2024).

[27] Y. Amma , K. Miura , S. Nagata , T. Nishi , S. Miyake , K. Miyazaki , M. Takashiri , Sci. Rep. 2022, 12, 21603. 36517530 10.1038/s41598-022-26108-y PMC9748887

[28] M. Nakatani , S. Fukamachi , P. Solís‐Fernández , S. Honda , K. Kawahara , Y. Tsuji , Y. Sumiya , M. Kuroki , K. Li , Q. Liu , Y. C. Lin , A. Uchida , S. Oyama , H. G. Ji , K. Okada , K. Suenaga , Y. Kawano , K. Yoshizawa , A. Yasui , H. Ago , Nat. Electron. 2024, 7, 119.

[29] A. F. A. Naim , A. G. El‐Shamy , Mater. Sci. Semicond. Process. 2022, 15, 107041.

[30] T. Low , M. Engel , M. Steiner , P. Avouris , Phys. Rev. B 2014, 90, 081408.

[31] J. Y. Oh , J. H. Lee , S. W. Han , S. S. Chae , E. J. Bae , Y. H. Kang , W. J. Choi , S. Y. Cho , J. O. Lee , H. K. Baik , T. I. Lee , Energy Environ. Sci. 2016, 9, 1696.

[32] X. Xia , S. Zhou , Y. Wang , Z. Zhang , Fundam. Res. 2023. 10.1016/j.fmre.2023.11.001 PMC12167882 40528986

[33] X. H. Tang , X. Z. Jin , Q. Zhang , Q. Zhao , Z. Y. Yang , Q. Fu , ACS Appl. Mater. Interfaces 2023, 15, 23286. 37139664 10.1021/acsami.3c02852

[34] W. Gao , N. Komatsu , L. W. Taylor , G. V. Naik , K. Yanagi , M. Pasquali , J. Kono , J. Phys. D: Appl. Phys. 2020, 53, 063001.

[35] M. Zhang , J. T. W. Yeow , Carbon 2020, 156, 339.

[36] K. Li , R. Yuasa , R. Utaki , M. Sun , Y. Tokumoto , D. Suzuki , Y. Kawano , Nat. Commun. 2021, 12, 3009. 34021142 10.1038/s41467-021-23089-w PMC8139987

[37] D. Suzuki , K. Li , K. Ishibashi , Y. Kawano , Adv. Funct. Mater. 2021, 31, 2008931.

[38] T. Cao , X. L. Shi , M. Li , B. Hu , W. Chen , W. D. Liu , W. Lyu , J. MacLeod , Z. G. Chen , eScience 2023, 3, 100122.

[39] P. Zhao , F. Yu , B. Wang , H. Zhao , C. Chen , D. Wang , P. Ying , Y. Wu , P. Li , B. Zhang , B. Liu , Z. Zhao , W. Hu , D. Yu , J. He , Z. Liu , B. Xu , Y. Tian , J. Mater. Chem. A 2021, 9, 4990.

[40] G. S. Hegde , A. N. Prabhu , J. Electron. Mater. 2022, 51, 2014.

[41] S. H. Jung , K. T. Kim , G. S. Lee , J. Y. Sun , D. W. Kim , Y. S. Eom , D. Y. Yang , J. Yu , J. M. Park , D. Y. Hyeon , K. I. Park , ACS Appl. Mater. Interfaces 2021, 13, 5125. 33478215 10.1021/acsami.0c20509

[42] Y. Ekubaru , T. Sugahara , K. Ibano , A. Suetake , M. Tsurumoto , N. Kagami , K. Suganuma , Adv. Mater. Technol. 2020, 5, 1901128.

[43] F. F. Jaldurgam , Z. Ahmad , F. Touati , A. A. Ashraf , A. Shakoor , J. Bhadra , N. J. Al‐Thani , D. S. Han , T. Altahtamouni , J. Alloys Compd. 2021, 877, 160256.

[44] T. Araki , K. Li , D. Suzuki , T. Abe , R. Kawabata , T. Uemura , S. Izumi , S. Tsuruta , N. Terasaki , Y. Kawano , T. Sekitani , Adv. Mater. 2024, 36, 2304048. 10.1002/adma.202304048 37403808

[45] R. Utaki , K. Li , M. Sun , Y. Tokumoto , Y. Kawano , in Proc. 44th Int. Conf. Infrared, Millimeter, and Terahertz Waves , IEEE, Paris 2019.

[46] K. Li , Y. Kinoshita , D. Shikichi , M. Kubota , N. Takahashi , Q. Zhang , R. Koshimizu , R. Tadenuma , M. Yamamoto , L. Takai , Z. Zhou , I. Sato , Y. Kawano , Adv. Opt. Mater. 2024, 12, 2302847.

[47] A. Sarbajna , A. G. Rösch , L. Franke , U. Lemmer , M. M. Mallick , Adv. Eng. Mater. 2023, 25, 2200980.

[48] Q. Meng , Y. Yang , S. Han , F. Meng , T. Liu , Polym. Compos. 2024, 45, 8176.

[49] K. Li , Y. Matsuzaki , S. Takahara , D. Sakai , Y. Aoshima , N. Takahashi , M. Yamamoto , Y. Kawano , Adv. Mater. Interfaces 2023, 10, 2300528.

[50] M. Murata , A. Yamamoto , Y. Hasegawa , T. Komine , A. Endo , J. Appl. Phys. 2017, 121, 014303.

[51] Y. Hu , Z. Du , Y. Yao , Y. Ma , Y. Li , Y. Xu , X. Zhao , T. Tao , B. Liang , S. Lu , J. Mater. Sci.: Mater. Electron. 2021, 32, 14368.

[52] H. Yamashita , K. Tsunoda , H. Nishino , S. Sato , J. Appl. Phys. 2021, 129, 173101.

[53] S. K. Amizhtan , A. J. Amalanathan , R. Sarathi , B. Srinivasan , R. L. Gardas , H. Edin , N. Taylor , IEEE Access 2022, 10, 18192.

[54] W. Li , Z. Ni , J. Wang , X. Li , IEEE Trans. Electron Devices 2019, 66, 2230.

[55] T. W. Shen , K. C. Chang , C.‐M. Sun , W. Fang , J. Micromech. Microeng. 2019, 29, 025007.

[56] J. Zhou , M. A. R. Miah , Y. Yu , A. C. Zhang , Z. Zeng , S. Damle , I. A. Niaz , Y. Zhang , Y. H. Lo , Opt. Express 2019, 27, 37056. 31873475 10.1364/OE.27.037056

[57] A. Varpula , K. Tappura , J. Tiira , K. Grigoras , O.‐P. Kilpi , K. Sovanto , J. Ahopelto , M. Prunnila , APL Photonics 2021, 6, 036111.

[58] R. Huang , X. Ji , Y. Liao , J. Peng , K. Wang , Y. Xu , F. Yan , Opt. Express 2019, 27, 23250. 31510606 10.1364/OE.27.023250

[59] M. Bauer , A. Rämer , S. A. Chevtchenko , K. Y. Osipov , D. Čibiraitė , S. Pralgauskaitė , K. Ikamas , A. Lisauskas , W. Heinrich , V. Krozer , H. G. Roskos , IEEE Trans. Terahertz Sci. Technol. 2019, 9, 430.

[60] X. Yang , A. Vorobiev , A. Generalov , M. A. Andersson , J. Stake , Appl. Phys. Lett. 2017, 111, 021102.

[61] Z. Xie , J. Wang , G. Lu , J. T. W. Yeow , Mater. Des. 2023, 235, 112383.

[62] J. Ding , H. Fang , Z. Lian , Q. Lv , J. L. Sunb , Q. Yan , Nanoscale 2018, 10, 10538. 29808184 10.1039/c8nr03108h

[63] K. Li , D. Suzuki , Y. Kawano , Adv. Photon. Res. 2021, 2, 2000095.

[64] K. Li , T. Araki , R. Utaki , Y. Tokumoto , M. Sun , S. Yasui , N. Kurihira , Y. Kasai , D. Suzuki , R. Marteijn , J. M. J. den Toonder , T. Sekitani , Y. Kawano , Sci. Adv. 2022, 8, eabm4349. 35544563 10.1126/sciadv.abm4349 PMC9094654

[65] Y. Oike , IEEE Trans. Electron Devices 2022, 69, 2757. 10.1109/ted.2021.3131116 PMC10168101 37168652

[66] R. Kawabata , K. Li , T. Araki , M. Akiyama , K. Sugimachi , N. Matsuoka , N. Takahashi , D. Sakai , Y. Matsuzaki , R. Koshimizu , M. Yamamoto , L. Takai , R. Odawara , T. Abe , S. Izumi , N. Kurihira , T. Uemura , Y. Kawano , T. Sekitani , Adv. Mater. 2024, 36, 2309864. 10.1002/adma.202309864 38213132

[67] N. Hareesha , J. G. Manjunatha , B. M. Amrutha , N. Sreeharsha , S. M. Basheeruddin Asdaq , Colloids Surf., A 2021, 626, 127042.

[68] X. Qi , H. Ha , B. Hwang , S. Lim , Appl. Sci. 2020, 10, 6983.

[69] S. Y. Tsai , C. C. Lin , C. T. Yu , Y. S. Chen , W. L. Wu , Y. C. Chang , C. C. Chen , F. H. Ko , Nanomaterials 2022, 12, 2428.35889654

[70] L. Duan , Y. Zhang , J. Zhao , J. Zhang , Q. Li , Y. Chen , J. Liu , Q. Liu , ACS Appl. Mater. Interfaces 2022, 14, 23951. 10.1021/acsami.2c02714 35537086

[71] H. Chen , Z. Wei , H. He , X. Zheng , K. S. Wong , S. Yang , Adv. Energy Mater. 2016, 6, 1502087.

[72] P. Fourmont , Y. Bai , F. X. Fortier , S. G. Cloutier , ACS Appl. Electron. Mater. 2022, 4, 5905.

[73] M. A. Shahbazi , L. Faghfouri , M. P. A. Ferreira , P. Figueiredo , H. Maleki , F. Sefat , J. Hirvonen , H. A. Santos , Chem. Soc. Rev. 2020, 49, 1253. 31998912 10.1039/c9cs00283a

[74] M. Zadan , A. Wertz , D. Shah , D. K. Patel , W. Zu , Y. Han , J. Gelorme , H. J. Mea , L. Yao , M. H. Malakooti , S. H. Ko , N. Kazem , C. Majidi , Adv. Funct. Mater. 2024, 34, 2404861.

