# Supplementary material for: All‐Solution‐Processable Hybrid Photothermoelectric Sensors with Carbon Nanotube Absorbers and Bismuth Composite Electrodes for Nondestructive Testing
Source: Small Sci. 2025 Feb 20;5(5):2400448. doi: 10.1002/smsc.202400448 (PMC12087774; doi:10.1002/smsc.202400448)
Supplement: Supplementary file 1 — Supplementary Material [file SMSC-5-2400448-s001.pdf]

Supporting Information

All-solution-processable hybrid photo-thermoelectric sensors with carbon nanotube absorbers and bismuth composite electrodes for non-destructive testing

Yuto Matsuzaki, Reiji Tadenuma, Yuto Aoshima, Minami Yamamoto, Leo Takai, Yukito Kon, Daiki Sakai, Norika Takahashi, Qi Zhang, Naoko Hagiwara, Ryo Koshimizu, Daiki Shikichi, Raito Ota, Sayaka Hirokawa, Meiling Sun, Yukio Kawano, and Kou Li**

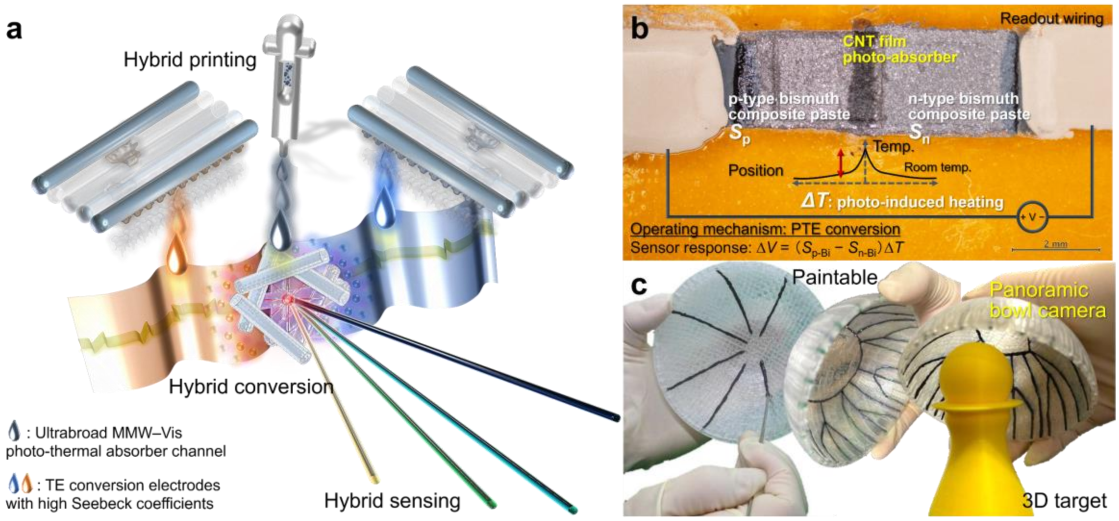

Figure 1. Conceptual diagram of this work.

a) Schematic of the presenting hybrid PTE sensor. b) Optical microscope image of the hybrid PTE sensor channel. c) Freely coatable and paintable configurations of the PTE sensor materials.

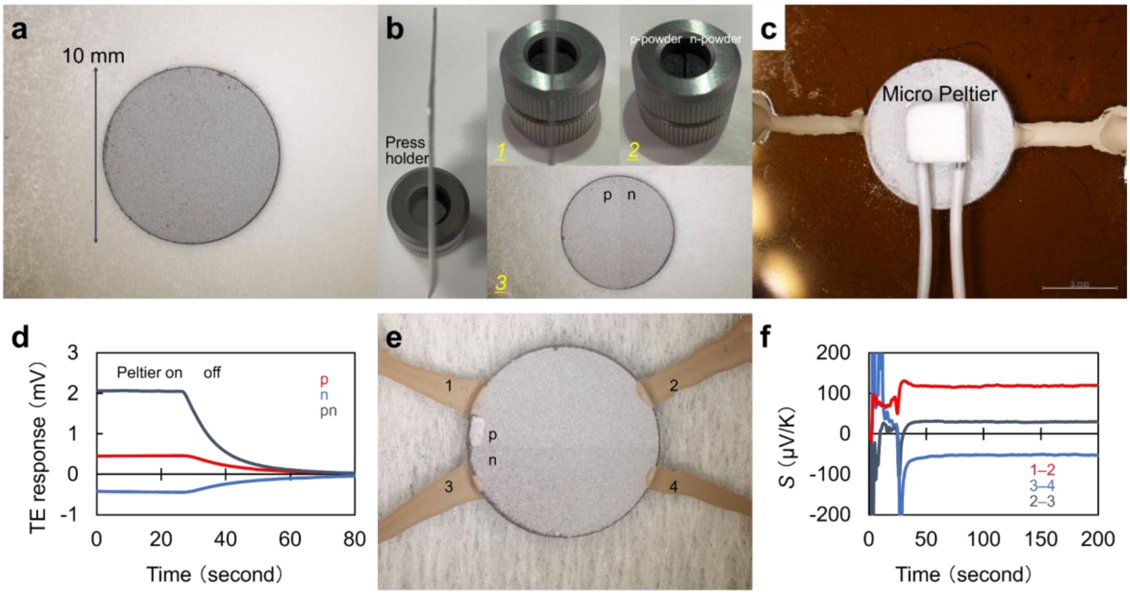

Figure S2. Fundamental characteristics of the pn-junction Bi$_{com}$ pellet.

a) Photograph of the Bi$_{com}$ pellet. b) Fabrication process of the pn-junction Bi$_{com}$ pellet. Compression pressure: 2 ton. c) Micro Peltier module mounted on the pn-junction of the pellet. d) Change in TE responses of Bi$_{com}$ pellets per constituent type. e) Photograph of the pn-junction Bi$_{com}$ pellet. f) Change in the Seebeck coefficient values of the Bi$_{com}$ pellet per probing position in (e).

Based on the obtained TE response signals and Seebeck coefficient values, this work simply aligns p-/n-type Bi$_{com}$ powders into the single pellet structure in an easy-to-operate fabrication process.

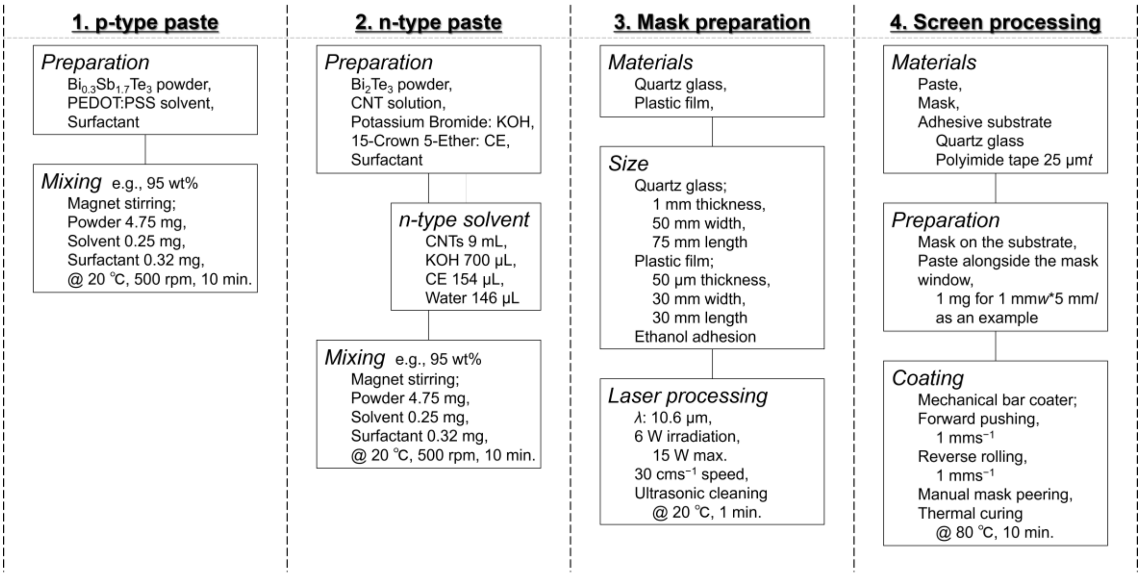

Figure S3. Experimental flowchart of Bi$_{com}$ paste preparations and the associated screen coating.

Mean diameter of powderlized Bi$_{com}$ materials: 10 µm. Magnetic stirrer: HS-1AN (AS One Co.).

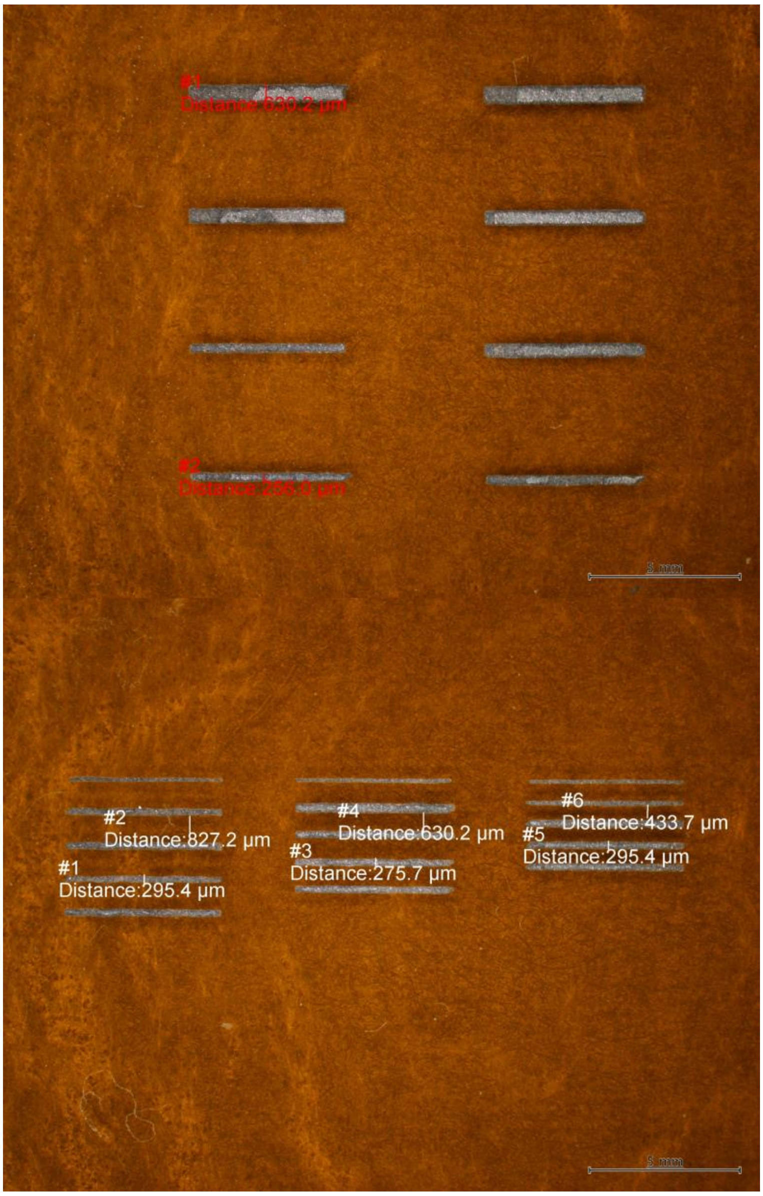

Figure S4. Simple line & space evaluation in screen coating of Bi_{com} pastes.

In the top optical microscope image, this work patterns Bi_{com} paste electrodes via screen coating with different line widths by controlling laser processing conditions for preparing mask windows. In the bottom optical microscope image, this work patterns Bi_{com} paste electrodes via screen coating with different spacing conditions.

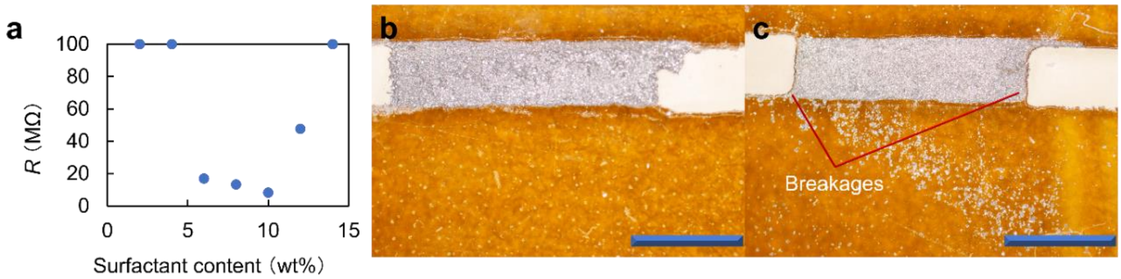

Figure S5. Mechanical robustness of the Bi$_{com}$ paste per surfactant content.
a) Change in electrical resistance values of the Bi$_{com}$ electrode after forming silver particle-binder resin mixed conductive wiring parts on its edges per surfactant content. b–c) Photographic comparison of the Bi$_{com}$ electrode-wiring interface with (b, 6 wt%) and without (c) the surfactant. Bi$_{com}$ powder content: 99 % (a–c), and scale bar: 2 mm (b–c).

In designing thin-film electronic devices, the use of stretchable wiring parts (derived from silver particle-binder resin mixed conductive paste) is indispensable for mechanical deformable configurations. Such wiring parts typically induce volumetric shrinkage characteristics during thermal annealing steps, affecting the counterpart materials as mechanical strains[S1]. Therefore, the mechanical robustness of Bi$_{com}$ pastes against thermal volumetric shrinkage behaviors from wiring parts is an essential factor in designing sensitive thin-film PTE sensor sheets. The obtained result indicates that the effective mixing of elastic surfactant agents in the presenting pastes enhances the mechanical robustness of the Bi$_{com}$ electrodes against forming steps of wiring parts. Specifically, the Bi$_{com}$ electrodes with the surfactant content range of 6–10 % are collectively conductive throughout wiring interfaces in (a), while their structures in lower ratio conditions (2–4 %) exhibit disconnections due to volumetric shrinkage-induced strains. Such disconnections within the device structure lead to crucial malfunctions of PTE sensors. From another viewpoint, the Bi$_{com}$ pastes with surfactant contents over 14 % are not suitable for the constituent part of the hybrid PTE sensor as electrically insulating elastic agents play a dominant role in the electrode formation. Based on the above, this work sets the surfactant content in the Bi$_{com}$ paste to 6 wt%, together with the findings in Figure 3i.

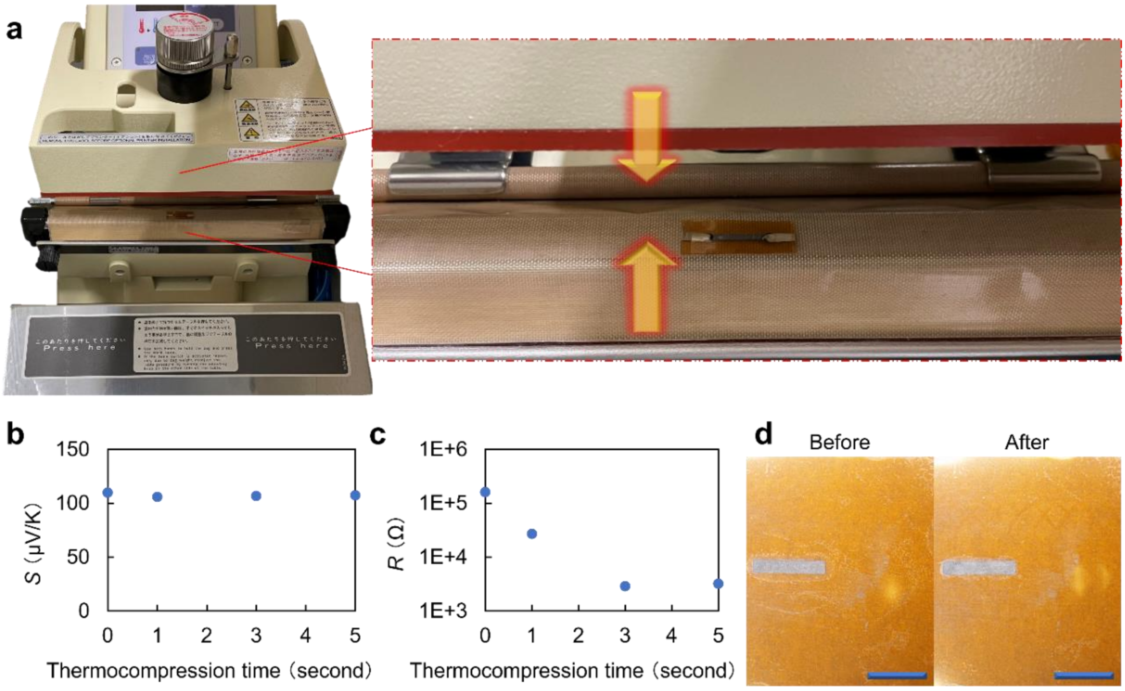

Figure S6. Changes in the fundamental characteristics of the Bi$_{com}$ paste by employing the thermocompression steps.

a) Photograph of the thermocompression setup. "Device fabrication" in the Experimental Section describes details of the equipment. b–c) Changes in vales of the Seebeck coefficient (b) and electrical resistance (c) for the hybrid PTE sensor against the thermocompression time. Bi$_{com}$ powder content: 90 %, and compression temperature: 100 °C (b–c). d) Photographic comparison of the paste-coated Bi$_{com}$ electrode before and after the thermocompression. Scale bar: 5 mm.

The obtained result indicates that the thermocompression strategy selectively attenuates the electrical resistance of the hybrid PTE sensor while maintaining the inherent Seebeck coefficient values of the Bi$_{com}$ paste (b–c). In short, the above trend infers that the morphological adsorption between the respective constituent materials via thermocompression steps maintains the inherent compositional characteristics of the Bi$_{com}$ paste. Simultaneously, the Bi$_{com}$ paste functions with sufficient physical robustness against mechanical stimulations during thermocompression steps, without exhibiting cracks (d). Based on these situations, this work includes the thermocompression in the device fabrication process of the hybrid PTE sensor.

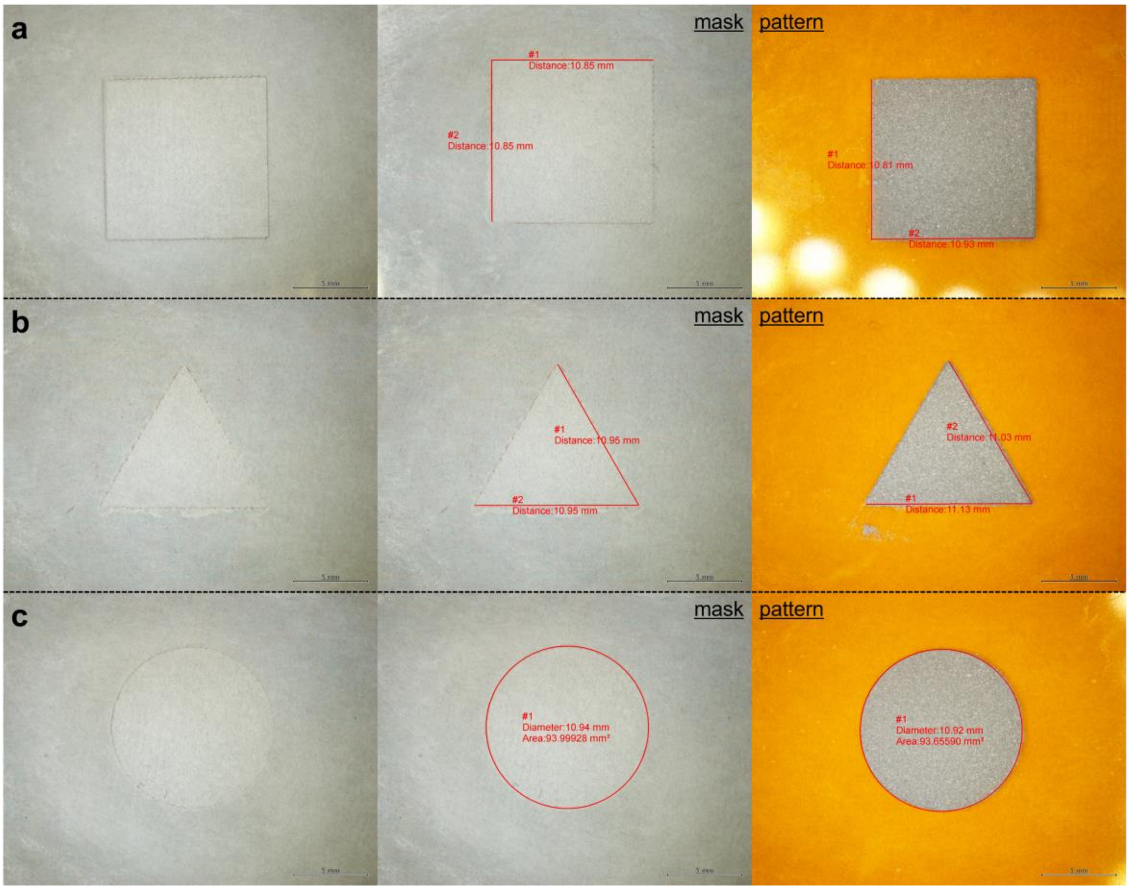

Figure S7. Shape and size evaluation of screen-coated thin-film electrodes from Bi_{com} pastes.

a–c) Photographs of laser-processed masks and their subsequent Bi_{com} paste patterns with different design shapes as follows: square (a), triangle (b), and circle (c). Bi_{com} powder content: 80 %.

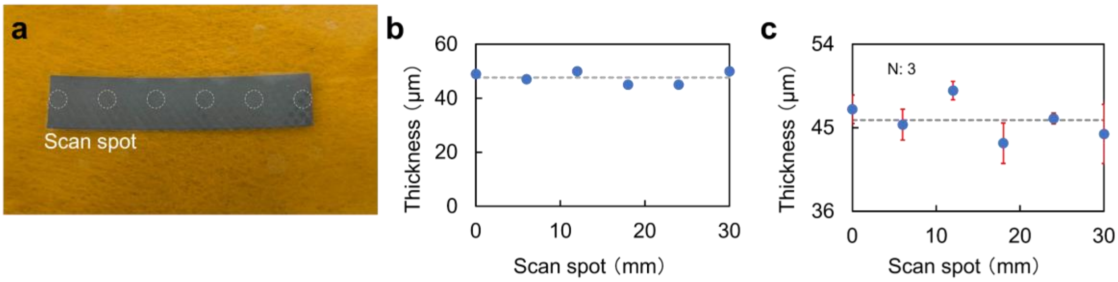

Figure S8. Thickness evaluation of screen-coated thin-film electrodes from Bi_{com} pastes. a) Photograph of the tested film (10 mm width and 30 mm length) after the thermocompression process in a 100 ℃ and 2-seconds condition. Bi_{com} powder content: 90 %. b) Thickness mapping of the film (a) with six scan spots in a channel length direction. In the obtained graph, a dotted line corresponds to an average value among six scanned thicknesses of the screen-coated Bi_{com} paste. c) Thickness distributions with three samples (a).

In these evaluations, this work employed a commercialized digital micro-meter (Coolant Proof MDC-PX, Mitutoyo Co.).

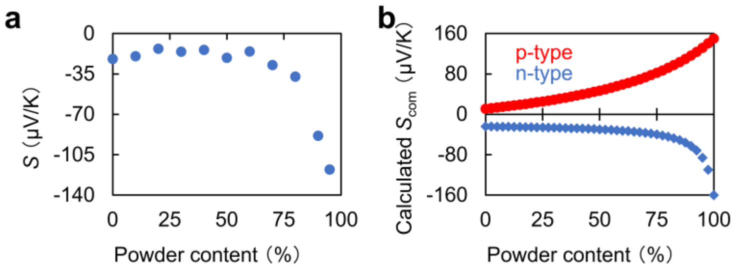

Figure S9. Numerical estimation of the composite Seebeck coefficient for Bi_{com} pastes. a) Seebeck coefficient mapping of n-type Bi_{com} pastes with different sample-sets from those in Figure 2k. b) Calculated composite Seebeck coefficient for p-/n-type Bi_{com} pastes (red-colored: p-type and blue-colored: n-type).

In handling composite materials, the following equation describes the total Seebeck coefficient:

$$S_{\mathrm{com}} = \frac{S_{\mathrm{a}}\sigma_{\mathrm{a}}v_{\mathrm{a}} + S_{\mathrm{b}}\sigma_{\mathrm{b}}v_{\mathrm{b}}}{\sigma_{\mathrm{a}}v_{\mathrm{a}} + \sigma_{\mathrm{b}}v_{\mathrm{b}}} \propto \frac{S_{\mathrm{a}}\frac{1}{\rho_{\mathrm{a}}}v_{\mathrm{a}} + S_{\mathrm{b}}\frac{1}{\rho_{\mathrm{b}}}v_{\mathrm{b}}}{\frac{1}{\rho_{\mathrm{a}}}v_{\mathrm{a}} + \frac{1}{\rho_{\mathrm{b}}}v_{\mathrm{b}}} \quad \text{S1}$$

where S_{com}, σ, ρ, and v respectively correspond to the total Seebeck coefficient of composite materials, the electrical conductivity/resistivity for their constituents, and the associated volumes[S2]. In the above estimation, this work sets constituents "a" and "b" for the composite material. For the employed constituents for pastes, this work also assigns the following material parameters as experimentally measured values: 150 μV/K for $S_{\text{p-Bicom}}$, –160 μV/K for $S_{\text{n-Bicom}}$, 11 μV/K for $S_{\text{PEDOT:PSS (p-type solvent)}}$, –24 μV/K for $S_{\text{n-CNTs (n-type solvent)}}$, 4.29 Ωcm for $\rho_{\text{p-Bicom}}$, 3.65 Ωcm for $\rho_{\text{n-Bicom}}$, 0.128 Ωcm for $\rho_{\text{PEDOT:PSS}}$, and 0.0151 Ωcm for $\rho_{\text{n-CNTs}}$.

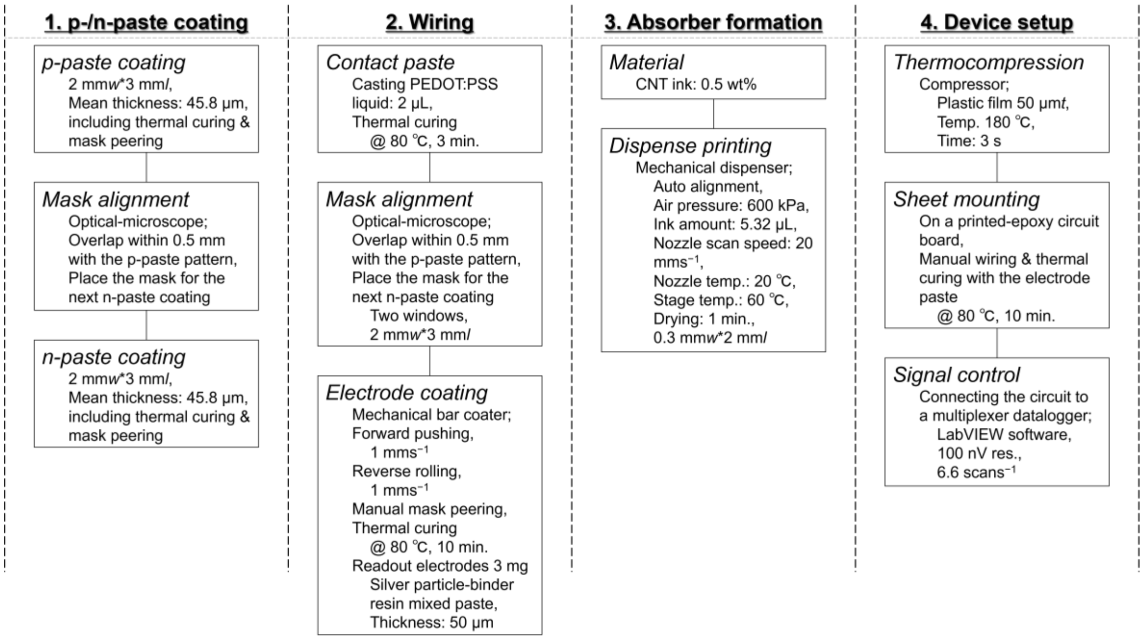

Figure S10. Experimental flow chart in fabricating the hybrid PTE sensor.

An example of the printed-epoxy circuit board: SSP-102 (1 mm pitch, Max. 40 Pin, Sunhayato Co.).

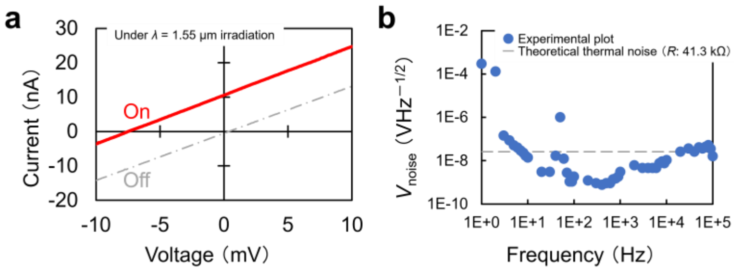

Figure S11. Fundamental PTE effect-induced behaviors of the presenting hybrid sensor. a) Current-Voltage characteristic of the hybrid PTE sensor with and without external irradiation. b) Noise spectral density of the device. Owing to the above zero-voltage-bias device operation, the hybrid PTE sensor functions with a lower limit of the theoretical thermal noise value[S3] during photo-detection operations. The peak value at 50 Hz originates from the power source of the measurement system (not from the device itself).

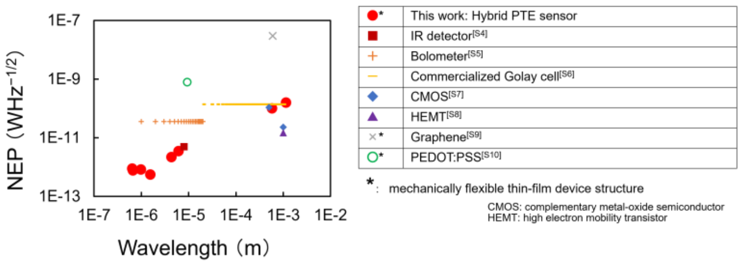

Figure S12. Photo-detection sensitivity (noise equivalent power: NEP values) mapping of the representative uncooled and nonvacuum sensor devices in ultrabroadband regions.

Table S1. Fundamental characteristics of the representative uncooled photo-sensors.

*CEP: cycling excitation process, a-Ge: amorphous germanium, a-Si: amorphous silicon, TiW: titanium-tungsten, TiN: tungsten nitride, AlGaN/GaN: aluminum-gallium nitride/gallium nitride, Mo_2C: molybdenum carbide

Type (Ref.)	Mechanism	Frequency	Material	Min. NEP	Structure
This work	PTE effect	*Ultrabroad* MMW–Vis	CNT inks, Bi_{com} pastes	560 $fWHz^{-1/2}$	Freely designable (Flexible thin-sheets, 2D rigid plates, 3D curvature surfaces, etc)
IR sensor (S4)	Photo-conductive, CEP	MIR	a-Ge, a-Si	5 $pWHz^{-1/2}$	Rigid solid-state
Bolometers (S5)	Temperature -induced current	*Broad* IR	TiW, TiN	36 $pWHz^{-1/2}$	Rigid solid-state
CMOS (S7)	High-freq. circuit	*Broad* THz	Silicon, Metal	23 $pWHz^{-1/2}$	Rigid solid-state
HEMT (S8)	High-freq. circuit	MMW	AlGaN/GaN	15 $pWHz^{-1/2}$	Rigid solid-state
Graphene (S9)	Plasmonic	Sub-THz	Graphene	3 $nWHz^{-1/2}$	Flexible sheet
PEDOT:PSS (S10)	PTE effect	FIR	Mo_2C/PEDOT :PSS	800 $pWHz^{-1/2}$	Flexible sheets
Microwire (S11)	Photodiode	*Broad* NIR–UV	Perovskite, Graphene	N.A.	Stretchable sheets

Table S2. Comparison of additional performances (response rate, time constant, and other specifications) in representative uncooled photo-detectors with the presenting hybrid PTE sensor: thin-film and solution-processable.

In the presenting comparison, the hybrid PTE sensor exhibits the following specifications: λ = 4.33 μm, ΔV = 38.8 mV, and P_{eff} = 3.31 μW, in a 10 mmφ-collimated beam-spot with a device electrical resistance of 41.3 kΩ (channel width: 1 mm).

Type (Ref.)	Response rate (VW^{-1})	Detectivity: D*	Time constant	Pixel integration
This work in ultra-broadband	11,700	$7.05^{*}10^{9}$ $cmHz^{1/2}W^{-1}$	154 ms	Tens of pixels at a 1 mm pitch with the minimum channel width of 256 μm
Fluorinated graphene in NIR (S12)	N.A.	N.A.	449 ms	Single pixel
Conductive polymer in NIR (S13)	0.768	N.A.	3 s	8 pixels at a 5 mm pitch with a channel width 10 mm
Micro-bolometer in MIR (S14)	45,000	$2.49^{*}10^{8}$ $cmHz^{1/2}W^{-1}$	6 ms	Within 10 pixels at a 100 μm pitch with a channel width of 100 μm
Pyroelectric in MIR (S15)	80,000	$5.13^{*}10^{8}$ $cmHz^{1/2}W^{-1}$	100 ms	Single pixel
Plasmonic absorber in FIR (S16)	410	$1.70^{*}10^{7}$ $cmHz^{1/2}W^{-1}$	176 μs	Three connected pixels in tens of μm-sq.
Optical antenna in THz (S17)	1,500	N.A.	N.A.	Sigle pixel
Vanadium dioxide in THz (S18)	5,000	$4.00^{*}10^{8}$ $cmHz^{1/2}W^{-1}$	13 ms	Single pixel
Field effect transistor in MMW (S19)	4,000	N.A.	N.A.	Single pixel

Based on the following comparison, the Bi_{com}-CNT hybrid paste-like PTE sensor sheet is advantageously available in an easy-to-operate all-solution-processable manner with freely attachable thin-film configurations (also, freely paintable and formable), while maintaining comparable fundamental photo-detection performances to those of representative optical devices in addition to the aforementioned ultrabroadband usability.

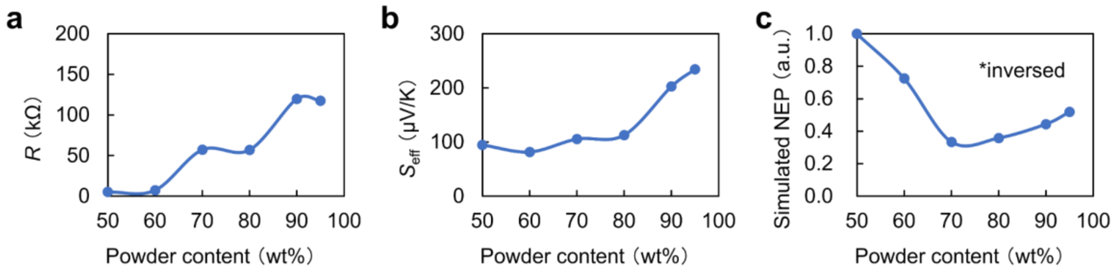

Figure S13. Photo-detection performance assessment of the hybrid PTE sensor per powder content of Bi$_{com}$-paste electrodes.

a) Change in resistance values of the hybrid PTE sensor per powder content in its Bi$_{com}$ paste-based electrodes. b) Change in the effective Seebeck coefficient of the pn-junction Bi$_{com}$ electrodes per powder content in their paste treatment. c) Simulated NEP values (inversed) of the hybrid PTE sensor per powder content in its Bi$_{com}$ paste-based electrodes.

Here, the following equation describes NEP values (performance index: photo-detection sensitivity) of PTE sensors:

$$\mathrm{NEP} = \frac{V_{\mathrm{Noise}}}{V_{\mathrm{Resopnse}}} = \frac{V_{\mathrm{Thermal\ noise}}}{S_{\mathrm{eff}} \times \Delta T} \times P_{\mathrm{eff}} \propto \frac{\sqrt{4k_{\mathrm{B}}TR}}{S_{\mathrm{eff}} \times \Delta T} \quad \text{S2}$$

where V_{Noise}, V_{Response}, $V_{\mathrm{Thermal\ noise}}$, P_{eff}, S_{eff}, ΔT, k_{B}, T, R are the noise voltage spectral density, normalized PTE direct-current voltage response, thermal noise voltage spectral density, effective Seebeck coefficient of the constituent material, photo-induced temperature gradient across the channel, normalized effective output power of irradiation onto the photo-detection interface, absolute temperature, electrical resistance of the device, respectively[S20]. Based on these preparations, this work simply simulates NEP values of the hybrid PTE sensor by taking the account of the experimentally obtained resistance and effective Seebeck coefficient values for the constituent material. The simulated trend (c) indicates that the use of Bi$_{com}$ paste electrodes with lower powder content conditions (< 70 %) leads to higher sensitive photo-detection operations of the hybrid PTE sensor by suppressing its electrical resistance values and the associated thermal noise signals. Contrary, the use of the hybrid PTE sensor with Bi$_{com}$ electrodes (powder content conditions over 90 %) facilitates higher-intensity signal acquisition in photo-detection operations, suitable for device coupling with miniature readout circuits as an example, by maximizing the effective Seebeck coefficient values in (b). Therefore, the presenting device design strategy is flexibly available depending on the purpose of PTE sensor operations.

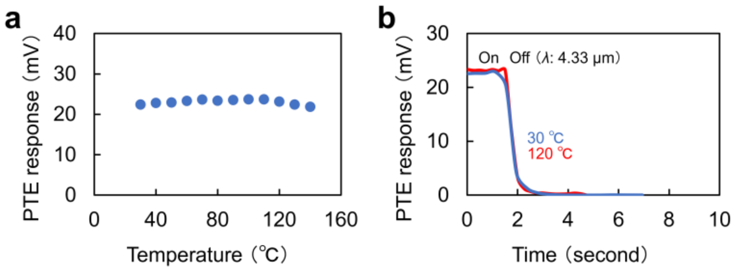

Figure S14. Photo-response signals of the hybrid PTE sensor under external irradiation with different temperature conditions in Figure 3e.

a) Intensity mapping. b) Comparison of on-off switching with the hybrid PTE sensor (at 30 ℃ and 120 ℃) in detecting external IR irradiation.

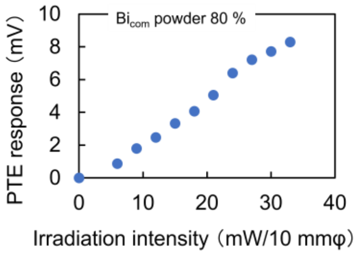

Figure S15. Change in response signals of the hybrid PTE sensor under photo-irradiation with different intensities.

λ: 1.55 μm. The hybrid PTE sensor exhibits a changing trend of photo-response signals proportional to external irradiation intensities. This behavior facilitates acquiring appropriate monochrome images with the hybrid PTE sensor in photo-measurements, appropriately reflecting respective changes in optical characteristics (transmission, reflection, absorption, and scattering) of monitoring targets.

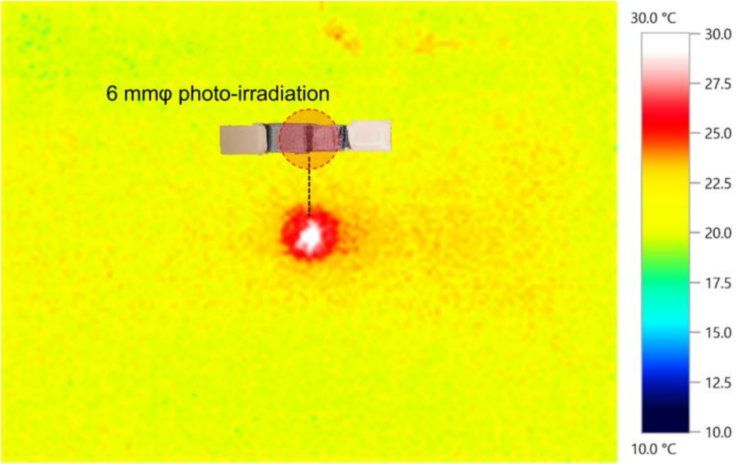

Figure S16. Thermography of the hybrid PTE sensor under external photo-irradiation. In the obtained thermography, the hybrid PTE sensor exhibits the highest temperature in its photo-detection interface and a gradual heat distribution (i.e., cooler) toward edges in the device length direction. According to such experimental behaviors, further finite element numerical simulations potentially facilitate deeper understanding in designing PTE sensor devices, being an essential next scope base on this work. Thermography: testo 868, Testo K.K.

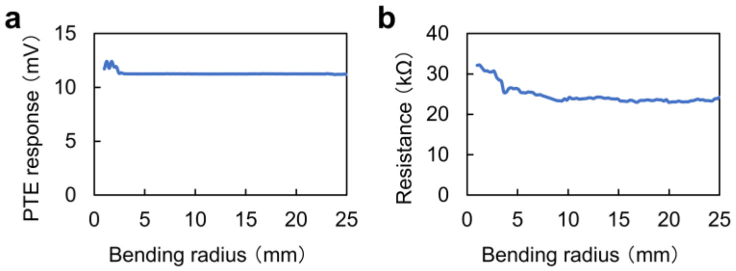

Figure S17. Optical stability of the hybrid PTE sensor against mechanical bending stimulations.

a) Stability in photo-detection response signals of the hybrid PTE sensor against mechanical bending across the device structure in Figure 3g. b) Changes in resistance values of the hybrid PTE sensor against mechanical bending across the device structure in Figure 3g.

In the presented advantageous optical stability of the hybrid sensor against mechanical deformations, constant PTE responses are dominantly effective. As the photo-detection response signal intensity of PTE sensors is proportional to their Seebeck coefficient values and temperature gradient across device structures[S11], the obtained result exhibits a stable trend against mechanical bending steps. For the remaining resistance values, the effective use of elastic surfactants in Bi_{com} paste by this work advantageously suppresses their changes within 30 % in a bending radius of 1 mm compared to the initial state in (b). Therefore, the presenting hybrid PTE sensor stably functions for photo-detection operations during mechanical deformations within the NEP change (proportional to the square root of resistance values) of 15 % in Figure 3g.

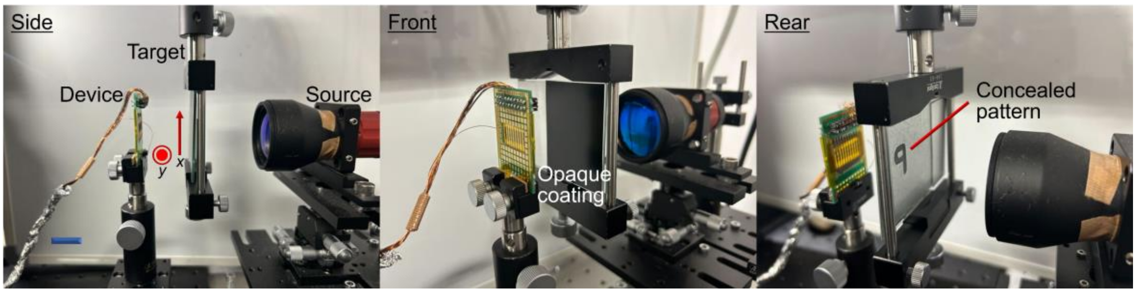

Figure S18. Experimental setup for optical measurements with multiple pixels-integrated hybrid PTE sensor array device (transmissive system).

Scale bar: 20 mm.

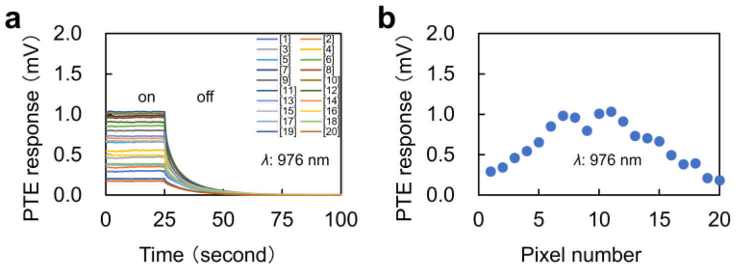

Figure S19. PTE responses for the transmissive non-destructive IR imaging with the hybrid PTE sensor array device in Figure 5a–c.

a) Photo-detection response signal distribution of the 20 pixels-integrated hybrid PTE sensor array device under full-face IR irradiation. b) Changes in response signal intensities of the hybrid PTE sensor array device for pixel positions (number) in (a). This work employs the obtained intensity ratio distribution as the response reference of the hybrid PTE sensor array device for the subsequent imaging signal calibration steps[S1] in Figure 5a–c.

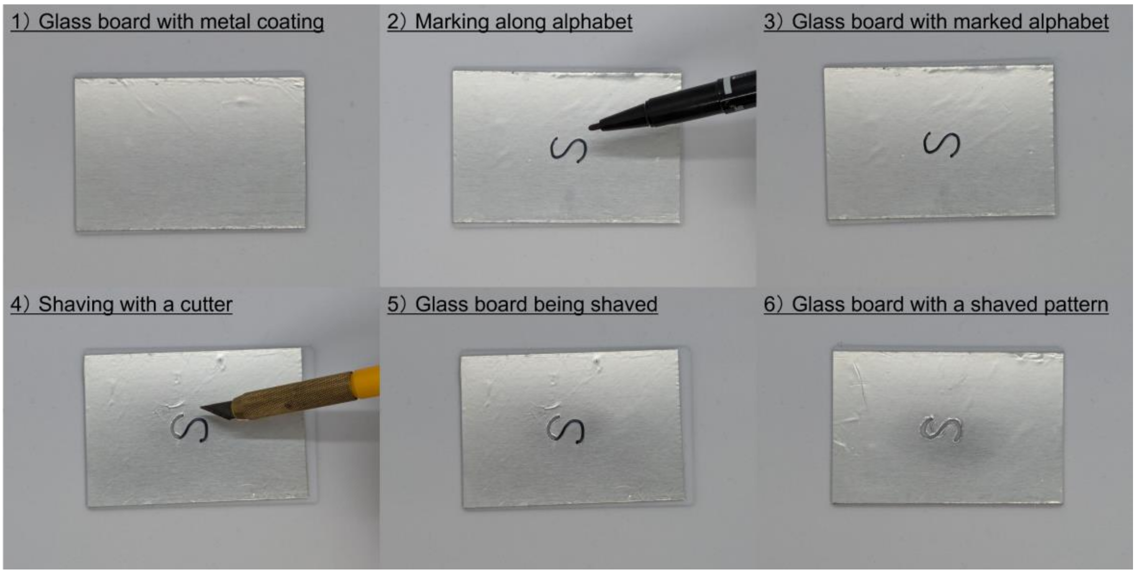

Figure S20. Preparation of the imaging target in Figure 5a–c.

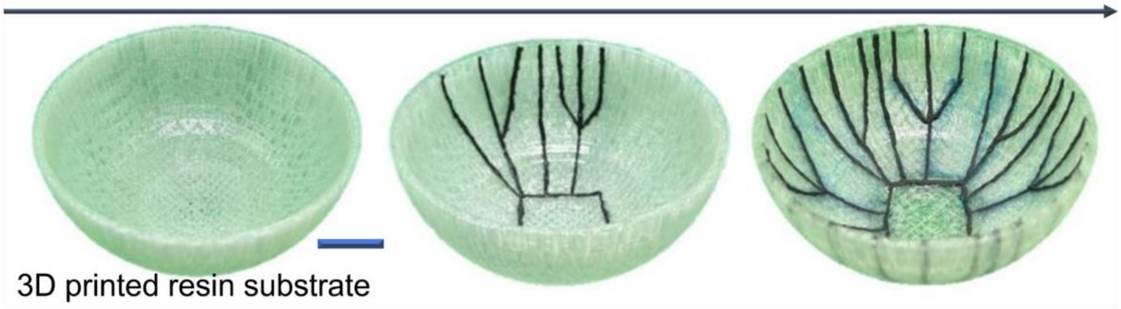

Figure S21. Fabrication process of the all-solution-processable hybrid PTE 3D panoramic bowl camera.

Scale bar: 20 mm.

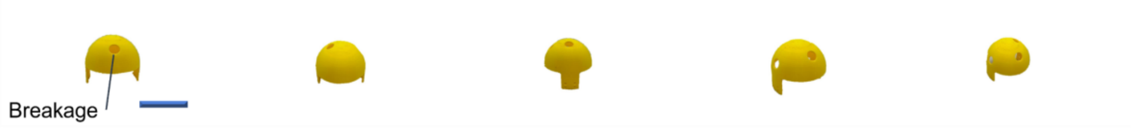

Figure S22. Imaging targe in Figure 5d–h.

Scale bar: 90 mm.

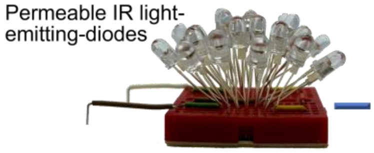

Figure S23. Panoramic IR source with 24 light emitting diodes (λ = 870 nm).

Scale bar: 10 mm.

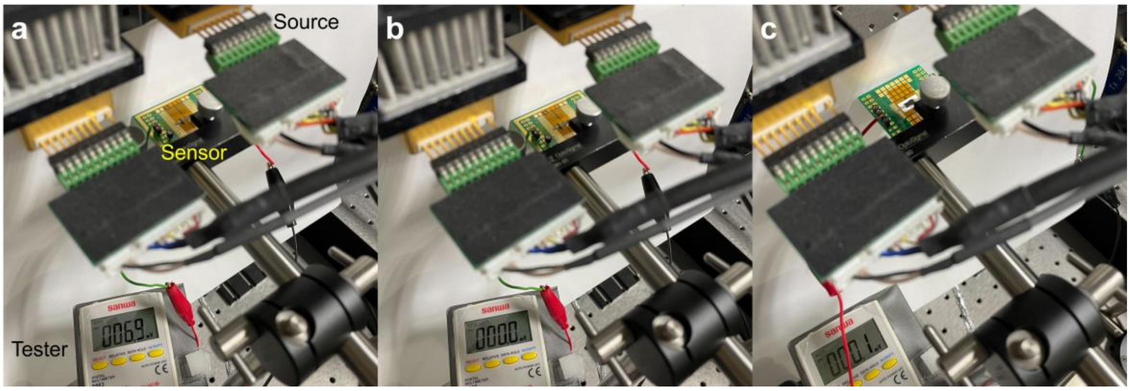

Figure S24. Device operations of PTE sensors under external photo-irradiation with a portable voltage tester in Figure 5k.

a–b) Experimental setup employing the hybrid PTE sensor with (a) and without (b) photo-irradiation. c) Experimental setup with the CNT film PTE sensor under photo-irradiation.

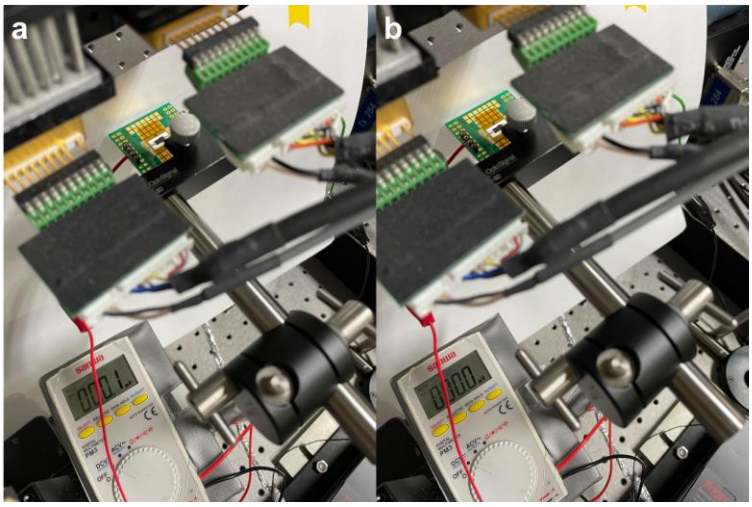

Figure S25. Photo-detection operation of the pn-junction CNT film PTE sensor under IR irradiation (in Figure 5) with the palm-sized digital tester.

a–b) Photographic views of the device operations with (a) and without (b) external photo-irradiation.

References

[S1] K. Li, Y. Matsuzaki, S. Takahara, D. Sakai, Y. Aoshima, N. Takahashi, M. Yamamoto, Y. Kawano, *Adv. Mater. Interfaces* **2023**, *10*, 2300528.

[S2] K. Li, D. Suzuki, Y. Kawano, *Adv. Photon. Res.* **2021**, *2*, 2000095.

[S3] X. Lu, L. Sun, P. Jiang, X. Bao, *Adv. Mater.* **2019**, *31*, 1902044.

[S4] J. Zhou, M. A. R. Miah, Y. Yu, A. C. Zhang, Z. Zeng, S. Damle, I. A. Niaz, Y. Zhang, Y. H. Lo, *Opt. Express* **2019**, *27*, 37056.

[S5] A. Varpula, K. Tappura, J. Tiira, K. Grigoras, O. P. Kilpi, K. Sovanto, J. Ahopelto, M. Prunnila, *APL Photon.* **2021**, *6*, 036111.

[S6] TYDEX LLC. https://www.tydexoptics.com/pdf/Golay_Detectors.pdf view date: 27th August, 2024.

[S7] R. Huang, X. Ji, Y. Liao, J. Peng, K. Wang, Y. Xu, F. Yan, *Opt. Express* **2019**, *27*, 23250.

[S8] M. Bauer, A. Rämer, S. A. Chevtchenko, K. Y. Osipov, D. Čibiraitė, S. Pralgauskaitė, K. Ikamas, Alvydas Lisauskas, Wolfgang Heinrich, Viktor Krozer, H. G. Roskos, *IEEE Trans. Terahertz Sci. Technol.* **2019**, *9*, 430.

[S9] X. Yang, A. Vorobiev, A. Generalov, M. A. Andersson, J. Stake, *Appl. Phys. Lett.* **2017**, *111*, 021102.

[S10] Z. Hie, J. Wang, G. Lu, J. T. W. Yeow, *Mater. Des.* **2023**, *235*, 112383.

[S11] J. Ding, H. Fang, Z. Lian, Q. Lv, J. L. Sunb, Q. Yan, *Nanoscale* **2018**, *10*, 10538.

[S12] J. Huang, C. Li, J. Liu, Y. Xie, *ACS Appl. Nano Mater.* **2024**, *7*, 14, 16215.

[S13] Y. Sozen, G. Ersu, T. Pucher, J. Quereda, A. Castellanos-Gomez, *Small Sci.* **2024**, *4*, 11, 2400063.

[S14] M. R. Hasan, A. Nikeghbal, S. Tran, A. Deshpande, M. U. Karkhanis, E. Pourshaban, *IEEE Sens. J.* **2024**, Early Access. doi: 10.1109/JSEN.2024.3427689

[S15] Z. Li, F. Liu, Y. Tang, X. Zhao, T. Wang, Z. Duan, W. Shi, F. Wang, J. Jiao, H. Luo, *J. Am. Ceram. Soc.* **2021**, *104*, 2, 995.

[S16] M. Dai, C. Wang, B. Qiang, Y. Jin, M. Ye, F. Wang, F. Sun, X. Zhang, Y. Luo, Q. J. Wang, *Nat. Commun.* **2023**, *14*, 3421.

[S17] X. Ren, Z. Ji, B. Chen, J. Zhou, Z. Chu, X. Chen, *Sensors* **2021**, *21*, 15, 5221.

[S18] M. W. Khan, O. Boyraz, *Int. J. Thermophys.* **2023**, *44*, 9.

[S19] I. Gayduchenko, S. G. Xu, G. Alymov, M. Moskotin, I. Tretyakov, T. Taniguchi, K. Watanabe, G. Goltsman, A. K. Geim, G. Fedorov, D. Svintsov, D. A. Bandurin, *Nat. Commun.* **2021**, *12*, 543.

[S20] K. Li, T. Araki, R. Utaki, Y. Tokumoto, M. Sun, S. Yasui, N. Kurihira, Y. Kasai, D. Suzuki, R. Marteijn, J. M. J. den Toonder, T. Sekitani, Y. Kawano, *Sci. Adv.* **2022**, *8*, eabm4349.
